# Comprehensive comparison of homologous recombination deficiency predictors in early-stage triple-negative breast cancer

**DOI:** 10.1186/s13058-026-02325-5

**Published:** 2026-06-25

**Authors:** Deborah F. Nacer, Srinivas Veerla, Iñaki Sasiain, Jari Häkkinen, Johan Vallon-Christersson, Maria Rossing, Serena Nik-Zainal, Anders Edsjö, Johan Staaf

**Affiliations:** 1 Division of Translational Cancer Research, Department of Laboratory Medicine, Lund University, Lund, Sweden; 2 Division of Oncology, Department of Clinical Sciences Lund, Lund University, Lund, Sweden; 3 Center for Genomic Medicine, Rigshospitalet, Copenhagen University Hospital, Copenhagen, Denmark; 4 Department of Clinical Medicine, Faculty of Health and Medical Sciences, University of Copenhagen, Copenhagen, Denmark; 5 Academic Department of Medical Genetics, School of Clinical Medicine & Early Cancer Institute, University of Cambridge, Cambridge, UK; 6 Department of Clinical Genetics, Pathology and Molecular Diagnostics, Skåne University Hospital, Region Skåne, Lund, Sweden; 7 Division of Pathology, Department of Clinical Sciences, Lund University, Lund, Sweden

## Abstract

**Background:**

Homologous recombination (HR) deficiency (HRD) is prevalent in ovarian, prostate, and specific subgroups of breast cancer, particularly triple-negative breast cancer (TNBC). Tumor HRD status can be inferred through DNA-based, RNA-based, functional, or image-based approaches. A comprehensive evaluation of the concordance and discordance among HRD prediction methods derived from these different data modalities has been lacking. In the present study, we systematically compared HRD classifications generated by seven distinct methods within a population-representative early-stage TNBC multi-omics cohort and contrasted them to an FDA-approved assay.

**Methods:**

A total of 235 patients from a reported population-based TNBC cohort from southern Sweden profiled by RNA-sequencing, whole genome sequencing, and with available RAD51 foci staining on tissue microarrays and digital whole slide H&E images were included. Seven different HRD classification methods were applied to available data, including sequencing-based (HRDetect and Classifier of HOmologous Recombination Deficiency, CHORD), copy number-based (scarHRD, and copy number signature 17), functional HR (RAD51-FFPE), mRNA-based, and image-based (DeepHRD) methods. Eighteen selected tumors were analyzed with the Myriad myChoice CDx assay for exploratory comparison. Survival analysis was performed using invasive disease-free survival as clinical endpoint in patients treated with adjuvant standard-of-care chemotherapy.

**Results:**

Overall, our results revealed substantial concordance across HRD assessment methods, alongside method-specific discordances attributable to differences in data preprocessing and, importantly, to training strategies that insufficiently account for the well-established clinical and molecular heterogeneity within breast cancer. Sequencing-based methods and scarHRD showed the greatest classification agreement, with discordance to some extent explained by aspects of inadequate tumor cell content, sequencing depth, and fundamental data processing steps (like segmentation). Discordance in mRNA- and image-based classifications appeared associated with molecular subtype features, suggesting that training cohort context may impact performance by incorporating signals (e.g., mRNA expression patterns) that are not specific to HRD status. Despite variation in HRD classification agreement, all seven methods displayed approximately similar prognostic performance in the subset of patients treated with adjuvant chemotherapy.

**Conclusions:**

Collectively, our findings underscore the necessity for rigorous optimization of data processing workflows and threshold definitions to ensure consistency, comparability, and reproducibility across HRD classification platforms.

**Supplementary material:**

The online version of this article (10.1186/s13058-026-02325-5) contains supplementary material, which is available to authorized users.

## Introduction

Triple-negative breast cancer (TNBC) is a clinical subgroup of breast cancer constituting ~ 10–20% of patients with primary disease defined by negativity for the estrogen receptor (ER), progesterone receptor, and *ERBB2/HER2* gene amplification [1, 2]. TNBC tumors display great molecular heterogeneity with respect to somatic alterations, mutational processes, the general tumor microenvironment (TME), and the specific tumor immune microenvironment (TIME) [3, 4]. A prominent genetic feature of TNBC is a high frequency of DNA repair deficiency, typically manifested as homologous recombination (HR) deficiency (HRD). It has been estimated that up to 60% of TNBC patients have HRD tumors based on large-scale whole genome sequencing (WGS) studies [5–8]. In TNBC, HRD is typically caused by disruption of genes involved in the HR pathway, most prominently *BRCA1*, *BRCA2, PALB2* and *RAD51C*, through various mechanisms including germline and/or somatic mutation and promoter hypermethylation [5, 6]. Importantly, tumors with HRD signatures have been shown to respond favorably to compounds that increase the demand on compensatory DNA repair pathways—typically nonhomologous end joining—such as DNA-damaging agents (e.g., platinum) and PARP inhibitors, irrespective of HR gene status [9–13], although the strongest clinical trial evidence remains primarily in ovarian cancer [14, 15]. Currently, HRD status is clinically actionable in ovarian cancer regardless of *BRCA1*/*BRCA2* status, but not yet in early-stage breast cancer.

Tumor HR status (HRD or HRP, for HR-proficient) can be estimated through multiple approaches, including DNA-based, RNA-based, functional assays, and image-based methods [16, 17]. HRD classification based on DNA analysis was initially performed using somatic copy number aberrations (SCNAs) or targeted panels (e.g., analyzing mutations in *BRCA1* and *BRCA2*), but more recently also through WGS. Examples of the former include the commercially FDA-approved Myriad myChoice CDx assay, academic implementations of similar HRD algorithms like scarHRD [18], and copy number (CN) signature approaches [19, 20]. These assays target the specific patterns of SCNAs (often referred to as “genomic scars” [21]) thought to be the result of genomic instability conferred by HRD. Other HRD classification methods more reliant on large-scale sequencing data include CHORD (Classifier of HOmologous Recombination Deficiency) [22], HRDetect [6], and more [23–25]. These sequencing-based classifiers rely on that HRD confers characteristic patterns of somatic mutations and structural variation (referred to as mutational signatures) in addition to SCNAs [7]. Gene expression-based approaches [26, 27] and more recently image-based artificial intelligence (AI) methods based on hematoxylin and eosin (H&E)-stained whole slide images (WSIs) [28–31] have also been reported, although the clinical usefulness of these methods remains to be validated. As an example of a recently proposed AI-based WSI method, the deep-learning tool DeepHRD delivers a computational HRD prediction score to guide treatment decisions in individual breast and ovarian cancer patients [28]. Finally, in situ approaches for assessing functional HRD, typically by analysis of RAD51 foci formation in formalin-fixed, paraffin-embedded (FFPE) tissue, have also been reported [32–34].

Given the number of available tools for HRD assessment, conflicting classifications for individual tumors are not unexpected. This discordance likely reflects differences in how HRD is defined and measured, as well as variation in the cancer types used to train each method [16, 35, 36]. Moreover, HRD classifiers based on genomic scars (SCNAs) or mutational signatures may not necessarily reflect the tumor’s current HRD status, as restoration of homologous recombination through mutations or promoter demethylation can occur during prolonged treatment [37, 38]. Functional HRD assays also face several practical limitations in clinical settings [17]. Understanding the concordance and discordance between HRD classifiers is therefore critical for clinical implementation, as it supports more informed interpretation of both definitive and ambiguous results and clarifies the limitations of each method.

In the present study we aimed to comprehensively study the agreement and disagreement between seven different HRD classifiers based on DNA, RNA, WSI, and functional assessment in early-stage treatment-naïve TNBC. We used a 235-sample TNBC cohort from South Sweden with matched WGS, RNA-sequencing, and promoter methylation data, FFPE tissue specimens, and digitalized H&E WSIs. In breast cancer, TNBC represents a useful case example due to its high HRD frequency caused by a high proportion of *BRCA1*/*BRCA2*-deficient tumors, the presence of distinct molecular phenotypes (e.g., the PAM50 classifier [39] Basal/nonBasal division and TNBC-specific mRNA subtypes known as TNBCtype [40]), and a heterogeneous TME/TIME [40–43]. Taken together, our results show both overall agreement among HRD classifiers and method-specific discordance arising from differences in data preprocessing and, importantly, from training approaches that may not fully capture the known clinical and molecular heterogeneity of breast cancer.

## Results

### HRD frequencies in TNBC by seven different algorithms

**Fig. 1:**
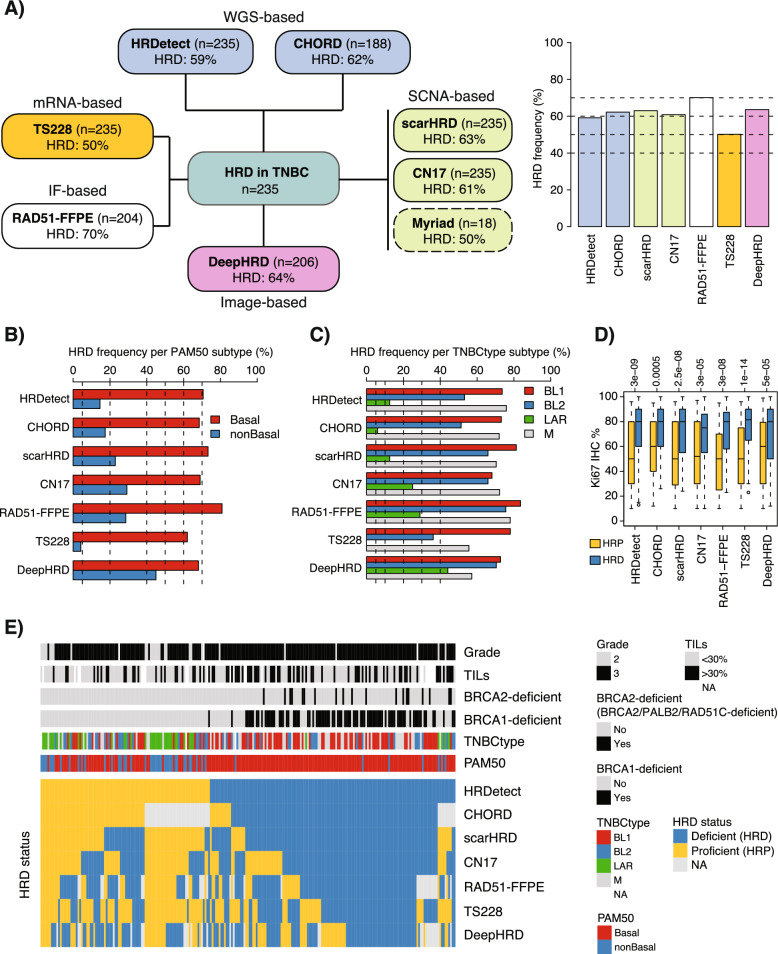
Methods overview and HRD frequencies in TNBC. **A** Outline of HRD methods compared and overall HRD frequency for each method. The n value between parentheses indicates the number of conclusive classifications from which the HRD frequency (percent) is computed. The Myriad myChoice CDx assay was only performed on a selected set of 18 cases not representative of the entire cohort and is therefore not shown on the panel on the right. **B** HRD frequency by the different methods in PAM50 Basal and nonBasal TNBC. **C** HRD frequency by the different methods in proposed mRNA-based TNBCtype subtypes of TNBC. **D** Ki67 immunohistochemistry (IHC) levels versus HRD class for each method. Top axis shows two-sided Wilcoxon’s test *p*-value for each method. **E** Overview of HRD classification concordance and discordance between methods versus molecular and clinicopathological classifications. TILs: tumor infiltrating lymphocytes

Seven main HRD classification methods were compared in this study (Fig. 1A). They represent five different approaches: i) sequence-based (HRDetect [6], CHORD [22]), ii) SCNA-based (CN17 [19], scarHRD [18]), iii) functional immunofluorescence (IF)-based (RAD51-FFPE [32]), iv) mRNA-based (a 228-gene mRNA-based nearest centroid classifier, hereon referred to as TS228 [27]), and v) image-based (DeepHRD [28]). Most samples (150/235, 64%) could be classified by all methods (Supplementary Figure S1), and HRD classifications per tumor are provided in Supplementary Table S1. We additionally obtained HRD status based on the clinically approved, FFPE-based, SCNA-based Myriad myChoice CDx assay for a small subset comprising 18 of the 235 tumors. Clinicopathological characteristics, patient inclusion and exclusion criteria, and individual treatment data for the 235-sample SCAN-B TNBC cohort are detailed in our previous study [5].

HRD frequencies obtained by the seven compared methods varied between 50 and 70% and are shown in Fig. 1A, together with the number of tumors classified by each method. Stratification by method type showed that the sequence- and SCNA-based methods had the most similar overall frequencies (approximately 60%), while the other methods varied more. Stratification of tumors by PAM50 subtype showed that the HRD frequency in Basal tumors was between 60–85% across methods, while notably lower in the nonBasal group (Fig. 1B). Looking within TNBC mRNA subtypes, most TNBCtype Basal-like 1 (BL1) tumors were classified as HRD (70–85%), followed by Mesenchymal (M), and Basal-like 2 (BL2) tumors (Fig. 1C). Luminal androgen receptor (LAR) tumors showed the lowest HRD frequency, in line with their predominant nonBasal PAM50 phenotype. Across all methods, tumors classified as HRD showed higher Ki67 immunohistochemistry (IHC) levels compared to HRP tumors on average (Fig. 1D), indicating higher tumor cell proliferation. For all methods, HRD tumors showed enrichment for *BRCA1*- or *BRCA2/PALB2/RAD51C*-deficiency (see Methods for definition) and for high tumor grade, but mixed levels of immune cell infiltration were observed (Fig. 1E). Notably, the level of enrichment of these variables appeared to vary more for non-DNA-based methods.

### General agreement between HRD predictors in primary TNBC

**Fig. 2:**
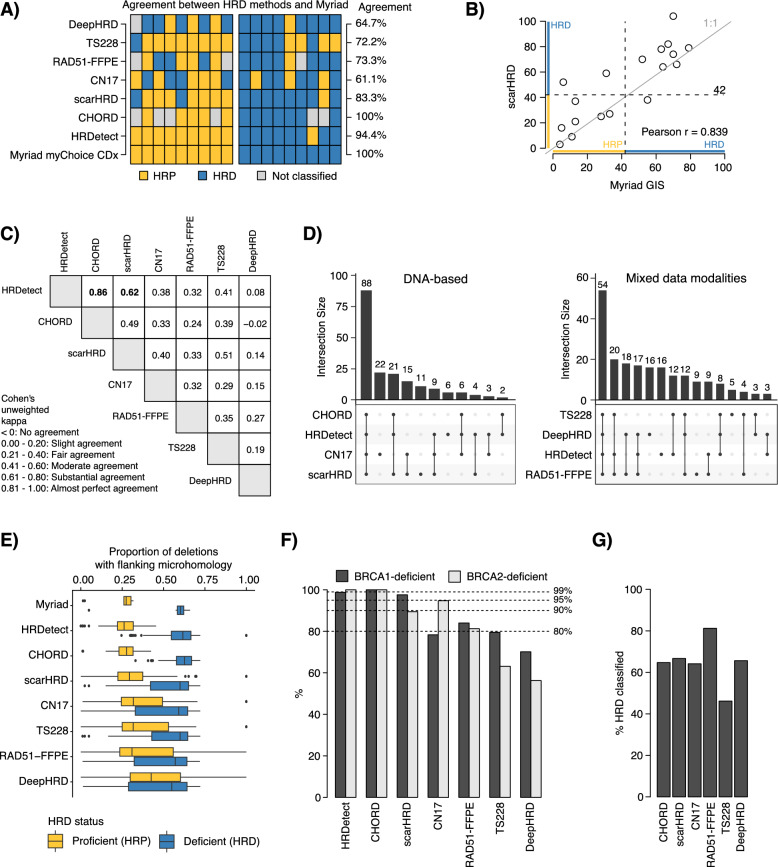
General agreement across HRD methods in primary TNBC. **A** Agreement between Myriad myChoice CDx HRD classification and HRD/HRP classes from the other methods as a percentage (right axis) and per sample (heatmap). **B** Scatter plot of Myriad myChoice CDx Genomic Instability Score (GIS) versus scarHRD scores and Pearson correlation coefficient. Dashed lines represent cutoffs used for HRD/HRP classification. **C** Unweighted Cohen’s kappa for classification comparison across the seven main HRD methods. Method combinations with substantial agreement are highlighted in bold. **D** Upset plots showing classification agreement for HRD tumors for different method combinations. **E** Proportion of deletions with flanking microhomology for HRD/HRP classes from all methods. **F** Percentage of *BRCA1*-deficient and *BRCA2/PALB2/RAD51C*-deficient (BRCA2-deficient) tumors classified as HRD by the seven main methods. **G** Percent of HRD-classified cases by different methods for 39 HRDetect HRD tumors without known *BRCA1/BRCA2*-deficiency. Boxplot elements correspond to: i) center line = median, ii) box limits = upper and lower quartiles, iii) whiskers = 1.5 × interquartile range

To assess classification agreement, we first compared all methods to the FDA-approved Myriad myChoice CDx HRD assay, for which HRD/HRP classifications were available for 18 selected tumors. This exploratory analysis, limited by sample numbers, demonstrated the highest agreement with CHORD (12/12, 100%) and HRDetect (17/18, 94%), followed by scarHRD (15/18, 83%) and the remaining methods (Fig. 2A). It is important to note that, while concordance was 100% between Myriad myChoice CDx and CHORD, 6/18 tumors (33%) could not be classified by CHORD. For HRDetect, the one tumor (PD31144a) with discrepant classification between Myriad myChoice CDx (HRD) and HRDetect (HRP) harbored both mismatch repair deficiency (MMRd) and *BRCA1* promoter hypermethylation. Presence of the MMRd mutational signature caused the original HRDetect algorithm used in Staaf et al. [5] to erroneously classify the tumor as HRP while visual inspection of, e.g., indel and structural variant signatures indicates HRD (see [5] and Supplementary Figure S1 for detailed WGS circos and SCNA plots). The scarHRD method uses an algorithm designed to approximate the Myriad Genomic Instability Score (GIS). As expected, a strong Pearson correlation was observed between the two metrics (Fig. 2B), although notable variability remained within this selected sample set. Importantly, this variability may further reduce agreement in binary classification between the two methods, especially in larger cohorts enriched for tumors with values near the GIS and scarHRD cutoffs of 42. With respect to classification concordance, it should be noted that DeepHRD is the only method performed on tissue sections derived from the same FFPE blocks used for the Myriad assay, whereas the RAD51-FFPE analysis was conducted on 1 mm tissue microarray (TMA) cores sampled from those FFPE blocks. Remaining methods used data derived from a separate fresh tissue sample from the tumor.

Next, we compared pairwise agreement among all methods except Myriad myChoice CDx using Cohen’s kappa. This analysis showed the highest kappa values between HRDetect and CHORD, followed by HRDetect and scarHRD (Fig. 2C). Remaining methods had typically lower kappa values, although moderate agreement was also noted between scarHRD and TS228. In more detail, Fig. 2D illustrates the greater HRD agreement between DNA-based methods (mainly HRDetect, CHORD, and scarHRD) compared to for instance HRDetect and mRNA-based (TS228), functional (RAD51-FFPE), or image-based (DeepHRD) classifications.

A distinct genetic feature of HRD tumors and particularly of *BRCA1*/*BRCA2*-deficient tumors is a high proportion of smaller deletions (< 100 bp) with flanking microhomology [6, 22]. Consistently, a difference in the proportion of deletions with flanking microhomology was observed between HRD and HRP tumors across methods, with the clearest separation for Myriad myChoice CDx, HRDetect and CHORD, followed by scarHRD (Fig. 2E). Here it should be noted that the separation with respect to proportions of deletions with microhomology is expected for HRDetect and CHORD, as this genetic feature is included in the two classifiers, in contrast to the remaining methods. For HRD classification methods not relying on mutational patterns or SCNAs, like RAD51-FFPE, TS228, and DeepHRD, the association between an HRD call and increased proportions of deletions with microhomology was less clear. Similarly, when focusing on tumors with *BRCA1*-deficiency (n = 83) or *BRCA2/PALB2/RAD51C*-deficiency (n = 19) defined by pathogenic somatic/germline variants or promoter hypermethylation, HRDetect, CHORD, and scarHRD most consistently identified such tumors as HRD (in line with their training), while the image-based DeepHRD method showed the least agreement (Fig. 2F). In contrast, for the 39 tumors classified as HRD by HRDetect without known *BRCA1*- and *BRCA2/PALB2/RAD51C*-deficiency, we found that most methods classified around two-thirds of these as HRD, with values ranging from 46% for TS228 to 81% for the RAD51-FFPE method (Fig. 2G).

Based on these general concordance results, we proceeded to analyze features of concordant and discordant HRD classifications for individual HRD classifiers. Due to a lack of gold standard HRD classification method, we used HRDetect as the reference classification given that it: i) represents a comprehensive WGS-based method including mutational and SCNA signatures, ii) could classify all tumors, iii) has been successfully applied to thousands of breast cancer samples from different academic institutions worldwide, demonstrating robustness versus different sampling and sequencing protocols and instruments [5, 6, 8–10, 44], and iv) showed high concordance with Myriad myChoice CDx in the exploratory analysis limited to 18 tumors.

### Features of concordant and discordant DNA-based HRD classifications

**Fig. 3:**
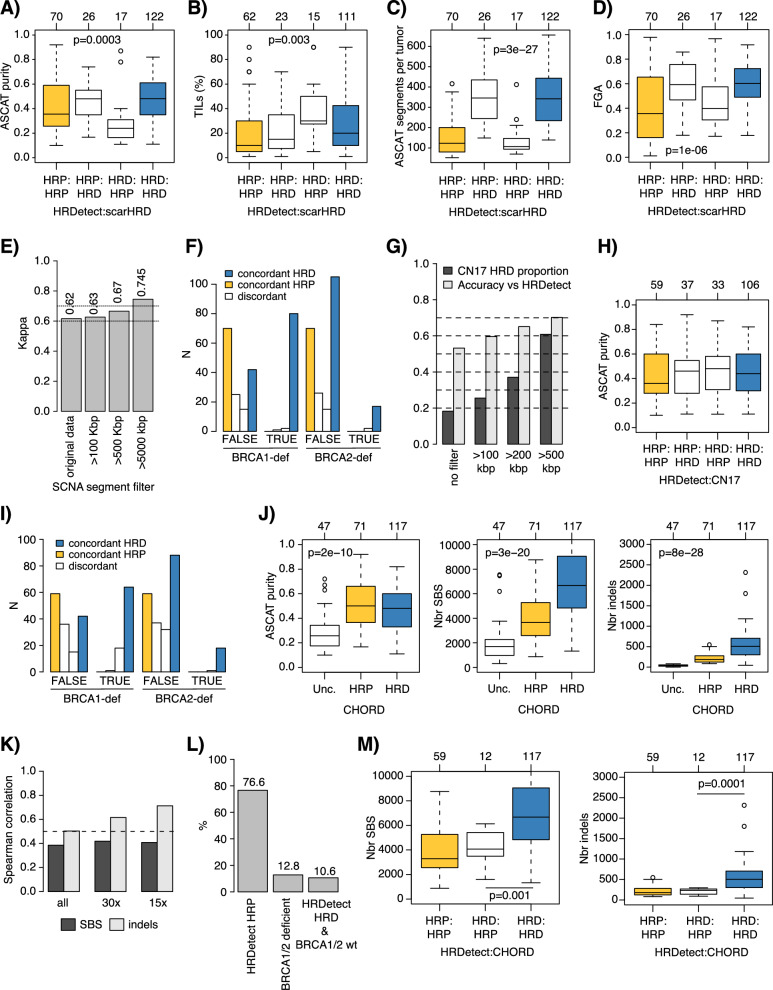
Concordance and discordance between scarHRD, CN17, and CHORD relative to HRDetect. **A** ASCAT tumor purity versus combinations of HRD classes obtained with HRDetect and scarHRD (HRDetect:scarHRD). Two-sided p-value computed using Kruskal–Wallis test. **B** Whole-slide H&E tumor infiltrating lymphocytes (TIL) estimates versus combinations of HRD classes for HRDetect:scarHRD. Two-sided p-value computed using Kruskal–Wallis test. **C** Number of ASCAT segments per tumor versus combinations of HRD classes for HRDetect:scarHRD. Two-sided p-value computed using Kruskal–Wallis test. **D** Fraction of the genome altered (FGA) by SCNAs (gain and loss) versus combinations of HRD classes for HRDetect:scarHRD. Two-sided p-value computed using Kruskal–Wallis test. **E** Agreement between HRDetect and scarHRD classifications (unweighted Cohen’s kappa) when applying different size filters to the original ASCAT SCNA segments. **F** Number of tumors (N) stratified by *BRCA1*-deficiency (BRCA1-def) or *BRCA2/PALB2/RAD51C*-deficiency (BRCA2-def) versus combinations of HRD classes for HRDetect:scarHRD. **G** CN17 HRD frequency (based on CN17 proportion > 0) and agreement with HRDetect for different ASCAT SCNA base pair segment size cutoffs. The > 500 Kbp represents the used CN17 classification. **H** ASCAT tumor purity versus combinations of HRD classes for HRDetect:CN17 using the > 500 Kbp CN17 classification. **I** Number of tumors (N) stratified by *BRCA1*-deficiency (BRCA1-def) or *BRCA2/PALB2/RAD51C*-deficiency (BRCA2-def) versus combinations of HRD classes for HRDetect:CN17. **J** ASCAT tumor purity estimates, number of SBSs and number of indels versus CHORD classes (Unc. = unclassified). Two-sided p-values computed using Kruskal–Wallis test. Y axes for SBSs and indels are truncated. **K** Spearman correlation values between ASCAT purity and number of SBSs and indels for all tumors, tumors sequenced at an average of 30x and 15x, respectively. **L** Proportion of the 47 unclassified CHORD tumors being called as HRP by HRDetect, *BRCA1/BRCA2*-deficient, or HRD by HRDetect but without known *BRCA1/BRCA2*-deficiency (wildtype, wt). **M** Number of SBSs and number of indels versus combined HRDetect and CHORD classes for 188 tumors classified by both methods. Two-sided p-values computed using Wilcoxon’s test for the indicated groups. Y axes for SBSs and indels are truncated. Boxplot elements correspond to: i) center line = median, ii) box limits = upper and lower quartiles, iii) whiskers = 1.5 × interquartile range. In boxplots, top axes indicate group sizes

Both scarHRD and CN17 classifications rely on segmented SCNA data, where tumor cell content affects the dynamic range of SCNAs (i.e., lower tumor cell content renders SCNA segments less distinct in amplitude). While allele-specific segmentation methods like ASCAT [45] aim to circumvent the issue of tumor cell content, we hypothesized that lower tumor cell content may still explain some discordance between scarHRD or CN17 and HRDetect. Indeed, tumors classified as HRD by HRDetect but HRP by scarHRD (HRD:HRP) showed lower ASCAT tumor purity estimates compared to remaining class combinations (Fig. 3A). Consistently, these discordant HRD:HRP tumors showed higher levels of tumor infiltrating lymphocytes (TILs) estimated by pathologists, indicating an immune-inflamed TIME (Fig. 3B). In contrast, HRP:HRD discrepant tumors showed higher number of ASCAT segments similar to concordant HRD tumors, although this did not translate to higher or lower proportion of SCNAs in the tumors overall (Fig. 3C and D). The former suggests that SCNA segmentation performance may have a role in scarHRD classification. To evaluate this hypothesis in more detail, we investigated how SCNA segment size affects scarHRD classification. This was done by filtering the original reported ASCAT data using three thresholds: i) removing segments ≤100 Kbp (retaining 81.2% of all 63,665 original segments), ii) ≤500 Kbp (retaining 65.6% of segments), or iii) ≤5000 Kbp (retaining 35.8% of segments). We then performed scarHRD analysis and HRD classification on each filtered set. Notably, concordance in HRD classification with HRDetect increased from 0.82 to 0.872 and the Cohen’s kappa from 0.62 to 0.745, respectively, when only longer segments were kept (Fig. 3E). Importantly, when tumors were stratified by *BRCA1*- and *BRCA2/PALB2/RAD51C*-deficiency, few deficient tumors were discordant between HRDetect and scarHRD (Fig. 3F). This suggests that discordant cases exhibit a SCNA phenotype less similar to the prototypical phenotype of *BRCA1/2* deficient tumors.

For CN17, we observed considerable variability in HRD frequency depending on the number and size of SCNA segments present in the data (Fig. 3G). Specifically, using the original ASCAT data from [5] resulted in an HRD frequency of 18.3% and a classification agreement of 53.2% compared to HRDetect (CN17 proportion cutoff > 0), while applying the 500 Kbp segment size filter resulted in an HRD frequency of 60.8% and an agreement of 70.2%. Together, these results suggest a considerable dependency of CN17 HRD classification on initial segmentation output. Based on filtered data (ASCAT segment size > 500 Kbp), discrepant HRDetect:CN17 cases did not appear associated with tumor purity and were mainly tumors without known *BRCA1*- and *BRCA2/PALB2/RAD51C*-deficiency (Fig. 3H, I).

While CHORD and HRDetect showed the highest classification agreement (93.6%), a notable feature of the CHORD analysis was that 20% (47/235) of investigated tumors could not be classified. These unclassified tumors were significantly associated with lower ASCAT tumor cell content, lower overall single base substitution (SBS) mutation counts, and lower indel counts (Fig. 3J). Overall, tumor purity was positively correlated with number of SBSs and indels, irrespective of sequence depth, in the 235-sample cohort (Fig. 3K). Unclassified CHORD tumors comprised a mix of *BRCA1*/*BRCA2*-deficient HRD tumors, HRD tumors (by HRDetect) without known *BRCA1*/*BRCA2*-deficiency, and HRDetect HRP tumors (Fig. 3L). Moreover, 70/235 (30%) tumors in the SCAN-B cohort were sequenced at a lower depth (~15x [5]), and the proportion of tumors unclassified by CHORD was higher in this 15x group compared to cases with > 30x coverage (35.7% versus 13.3%, respectively). Together, these results suggest that CHORD classification is affected by interdependent tumor cell content and sequence depth. For tumors with both CHORD and HRDetect classifications (n = 188), CHORD classified 12 HRDetect HRD tumors as HRP (HRD:HRP). None of these 12 tumors had *BRCA1*- or *BRCA2/PALB2/RAD51C*-deficiency, and there was no difference in ASCAT tumor purity compared to concordantly classified HRP or HRD tumors (Kruskal–Wallis *p* = 0.21). The 12 discrepant tumors did, however, show significantly lower mutational burden (SBS and indels) compared to concordantly classified HRD tumors (Fig. 3M), despite that 8/12 (67%) tumors were sequenced with > 30x coverage.

### Features of concordant and discordant functional RAD51 HRD classifications

**Fig. 4:**
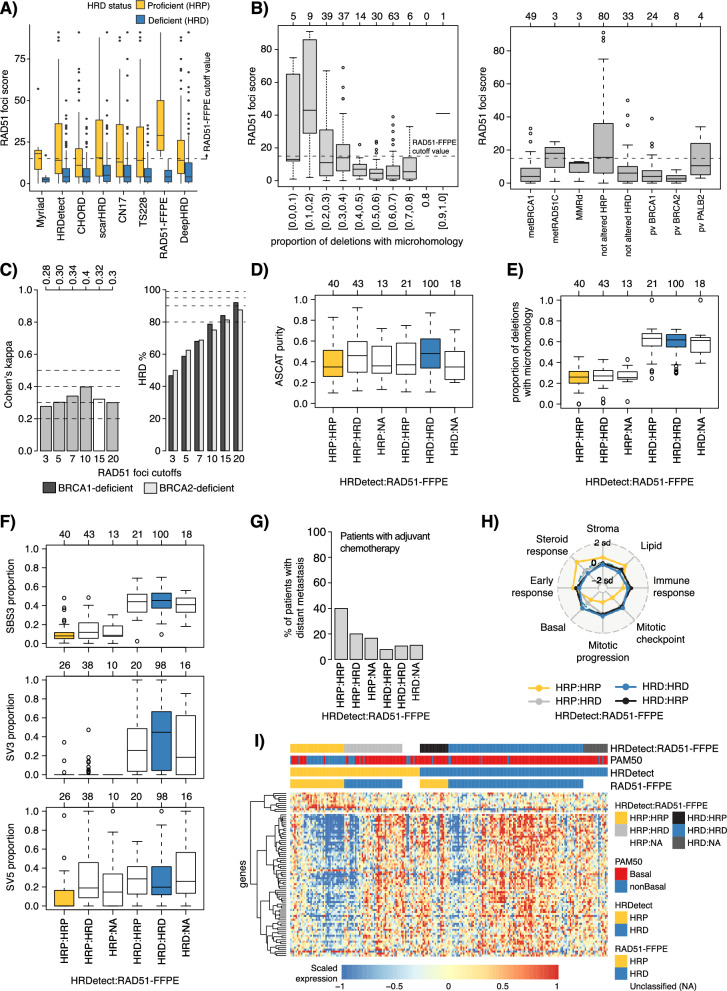
Functional HRD and concordance and discordance for the RAD51-FFPE test. **A** RAD51 foci scores versus HRD/HRP status for analyzed HRD methods. **B** RAD51 foci scores versus stratified proportions of deletions with microhomology in the total tumor cohort (left) and versus different HRD inactivation mechanisms and MMRd (right). Met: methylated promoter; pv: pathogenic variant (germline or somatic). **C** Left: agreement kappa values of RAD51-FFPE classification based on different RAD51 foci score cutoffs. A white bar indicates the original classification using a cutoff of 15. Right: HRD frequency in *BRCA1*- and *BRCA2*-deficient tumors for different RAD51 foci score cutoffs. **D** Tumor purity estimated by ASCAT versus combined HRDetect and RAD51-FFPE groups (HRDetect:RAD51-FFPE). White boxes indicate discordant HRD calls between the two methods. **E** Proportions of deletions with microhomology versus HRDetect:RAD51-FFPE groups. **F** Exposure (proportion) of SBS3, SV3 and SV5 signatures versus HRDetect:RAD51-FFPE groups. **G** Proportion of patients with reported distant metastases among those treated with adjuvant chemotherapy versus HRDetect:RAD51-FFPE groups. **H** Radar plot of scaled gene expression rank scores for eight biological metagenes for concordant and discordant HRDetect:RAD51-FFPE class combinations in all tumors. Rings correspond to standard deviations (SD) of 2, 0, and -2. **I** Heatmap of median-centered, log2-transformed FPKM (fragments per kilobase million) data for 82 genes (rows) associated with DNA repair per sample (columns). Clustering was performed using Pearson correlation and Ward.D linkage. Annotation tracks show HRDetect:RAD51**-**FFPE groups, PAM50 status, and individual HRDetect and RAD51**-**FFPE HRD status. Boxplot elements correspond to: i) center line = median, ii) box limits = upper and lower quartiles, iii) whiskers = 1.5 × interquartile range. In boxplots, top axes indicate group sizes. In panels D-G, tumors that could not be classified by RAD51-FFPE (NA) are included for reference, stratified by their HRDetect status

A total of 204 tumors could be classified by the functional RAD51-FFPE test [32] using data from Kramer et al. [46]. In this test, RAD51-FFPE foci scores represent the percentage of geminin-positive cells with two or more RAD51 foci, with lower scores indicating HRD. As the RAD51-FFPE test represents the only functional HRD test in the comparison, foci scores were first compared between HRD/HRP classifications from all other methods (Fig. 4A). While this comparison demonstrated that tumors classified as HRD by all methods showed lower number of RAD51 foci, consistent with functional HRD, notable overlap existed in the lower foci range between HRD and HRP tumors. To explore this overlap in more detail we compared the functional RAD51 foci score to the proportion of deletions with microhomology, representing a characteristic genetic “scar” of *BRCA1*/*BRCA2*-deficient HRD tumors (Fig. 2E), finding a substantial drop in number of called RAD51 foci at a proportion of microhomology > 20%, which was further enhanced for proportions > 40% (Fig. 4B). Analysis of RAD51 foci scores versus different proposed HRD inactivation mechanisms confirmed that tumors with such mechanisms showed typically lower RAD51 scores, but also that a substantial number of HRD tumors without a known mechanism displayed low scores, as well as a proportion of HRP tumors (Fig. 4B). Building on this, we analyzed how different RAD51 score cutoffs affected the kappa agreement value relative to HRDetect classification, finding that reducing the score cutoff from equal to or above 15 to 10 increased the kappa value from 0.32 to 0.4, but that further reductions did not improve agreement (Fig. 4C). In opposite, decreasing the RAD51 foci cutoff reduced the proportion of *BRCA1*- and *BRCA2*-deficient tumors called as HRD by the RAD51-FFPE assay (Fig. 4C).

To further explore concordance and discordance between the RAD51-FFPE and the HRDetect classifications we analyzed combined HRD/HRP classes for differences in estimated tumor purity (Fig. 4D), proportions of deletions with microhomology (Fig. 4E), and exposures to mutational signatures characteristic of HRD tumors like single base substitution signature 3 (SBS3) and structural rearrangement signatures 3 and 5 (SV3 and SV5, respectively) (Fig. 4F). These analyses did not support that discordance was due to differences in tumor cell content for the WGS method, or that tumors that were discrepantly classified as HRD by HRDetect but HRP by RAD51-FFPE (HRD:HRP) appeared to have less exposure to BRCA1/BRCA2/HRD-associated mutational patterns. Moreover, when we compared the number of distant metastases reported in patients treated with adjuvant chemotherapy stratified by the combined classifications, the discrepant HRD:HRP group showed the lowest proportion of relapses followed by the concordant HRD:HRD group (Fig. 4G). In contrast, the concordant HRP:HRP group showed the most relapses. Together, these results do not provide clear support for discordance between the methods being associated with differences in patient outcome.

To further investigate concordance and discordance between HRDetect and RAD51-FFPE we used matched RNA-sequencing data. First, we analyzed differences in broader transcriptional programs assessed by rank scores for eight reported biological metagenes in breast cancer (termed lipid, immune response, mitotic progression, mitotic checkpoint, steroid response, basal, early response, and stroma) [47]. In this analysis, concordant HRP:HRP tumors appeared to show most differences compared to other groups, while discordant HRD:HRP tumors did not appear different from concordant HRD:HRD tumors (Fig. 4H, Supplementary Figure S2). Notably, the observed differences in expression of biological metagenes in HRP:HRP tumors are likely associated with that the HRP:HRP group is dominated by PAM50 nonBasal tumors (29/40, 72.5%), and such tumors typically show for instance higher values of the steroid response metagene and lower values of the basal and proliferation-associated metagenes [41]. We also performed a differential gene expression analysis targeting 219 genes associated with DNA repair deficiency (Supplementary Table S2). Of these 219 genes, 214 were available in our RNA-sequencing data and 82 of them showed a false discovery rate (FDR)-adjusted Kruskal–Wallis *p*-value < 0.05 when tested across the combined HRDetect and RAD51-FFPE groups. Visualization of these genes showed that the main expression difference appeared to be between the concordant HRP:HRP tumors and the remaining groups (Fig. 4I). This conclusion is consistent with the previously observed differences in biological metagenes and the enrichment of nonBasal TNBC tumors in this group. Included in the 82 gene list were *RAD51* and *BRCA1*, and while *RAD51* showed lowest expression in concordant HRP:HRP tumors, *BRCA1* showed elevated expression in HRP:HRD and HRP:NA (i.e., without RAD51-FFPE classification) tumors (Supplementary Figure S2).

### Features of concordant and discordant RNA-based HRD classification

**Fig. 5:**
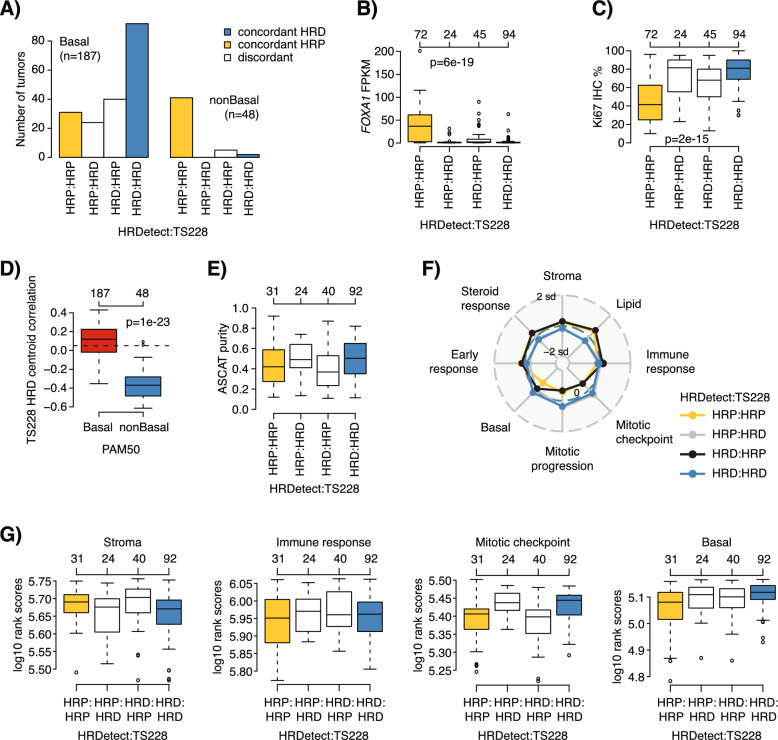
Concordance and discordance between the mRNA-based HRD predictor and HRDetect. **A** Number of concordant and discordant tumors for combined HRD/HRP classes by HRDetect and TS228 (HRDetect:TS228) stratified by PAM50 Basal/nonBasal subtypes. **B** *FOXA1* mRNA expression (FPKM) versus HRDetect:TS228 classes. Two-sided p-value calculated using Kruskal–Wallis test. **C** Ki67 immunohistochemistry (IHC) percentage levels versus HRDetect:TS228 classes. Two-sided p-value calculated using Kruskal–Wallis test. **D** Correlation values to the TS228 HRD centroid used for sample classification versus PAM50 subtypes. Two-sided p-value calculated using Wilcoxon’s test. **E** ASCAT WGS tumor purity for concordant and discordant class combinations in PAM50 Basal tumors. **F** Radar plot of scaled gene expression rank scores for eight biological metagenes for concordant and discordant class combinations in PAM50 Basal tumors. Sd = standard deviation. **G** Corresponding boxplots of log10-transformed gene expression rank scores for four selected metagenes in PAM50 Basal tumors, stratified by concordant and discordant classes (HRDetect:TS228). Boxplot elements correspond to: i) center line = median, ii) box limits = upper and lower quartiles, iii) whiskers = 1.5 × interquartile range. In boxplots, top axes indicate group sizes

In contrast to DNA-based HRD assays that rely on somatic alterations of only malignant cells, bulk RNA-based classifiers such as TS228 are derived from gene expression profiles from both malignant and non-malignant cell populations of typically unknown proportions and variable transcriptional magnitude. A further potential confounder in the training and application of RNA-based classifiers is the presence of underlying molecular subtypes characterized by distinct transcriptional programs in the cohorts used. In the study by Jacobson et al. [27], the TS228 centroids were notably derived by training in 572 TCGA tumors of both mixed clinical and molecular subtypes. Here, TNBC may be divided into mRNA-based subtypes [40, 42, 43], where for instance the PAM50 Basal and nonBasal division is also strongly associated with different HRD frequency [5] (Fig. 1). Therefore, we first analyzed classifier agreement between HRDetect and the TS228 mRNA classifier within PAM50 subtypes, finding that discordant cases are mainly present within the Basal subtype (Fig. 5A). Notably, while Cohen’s kappa was 0.41 between HRDetect and TS228 in the total cohort, it decreased to 0.24 in the 187 PAM50 Basal tumors alone. To further explore TS228 classification with underlying PAM50 Basal/nonBasal molecular subtypes we analyzed *FOXA1* mRNA expression against classifier agreement. *FOXA1* is a key transcription factor essential for ER attachment to chromatin and the subsequent transcriptional induction of luminal genes in breast cancer cells [48], with expression in TNBC strongly associated with a nonBasal phenotype [41]. A significant expression difference was observed between HRD classes, with tumors concordantly classified as HRP by HRDetect and TS228 (HRP:HRP) showing high *FOXA1* expression (Fig. 5B). We also analyzed IHC levels of the proliferation marker Ki67, observing less proliferation in HRP:HRP tumors (Fig. 5C). Finally, Pearson correlation values for the TS228 HRD centroid were significantly lower in nonBasal compared to PAM50 Basal tumors (Fig. 5D). Together, these observations suggest that the TS228 HRP classification in TNBC is skewed towards a nonBasal phenotype.

To explore concordance and discordance more specifically in Basal tumors we first analyzed whether ASCAT tumor purity differed between combined classes, however without finding any significant pattern (Fig. 5E). This inconclusive observation prompted us to investigate broader transcriptional programs assessed by rank scores for the eight biological metagenes [47], but no consistent metagene differences were observed for the two discordant classes (HRD:HRP and HRP:HRD) (Fig. 5F, G, Supplementary Figure S3). Consistent with these inconclusive findings, differential gene expression analysis of the 228 genes in the TS228 signature versus HRDetect status identified 114 differentially expressed genes in the total cohort, but only two genes (*BRCA1* and *TK1*) in Basal tumors specifically (FDR-adjusted Wilcoxon’s test *p* < 0.05).

### Features of concordant and discordant image-based HRD classification

**Fig. 6:**
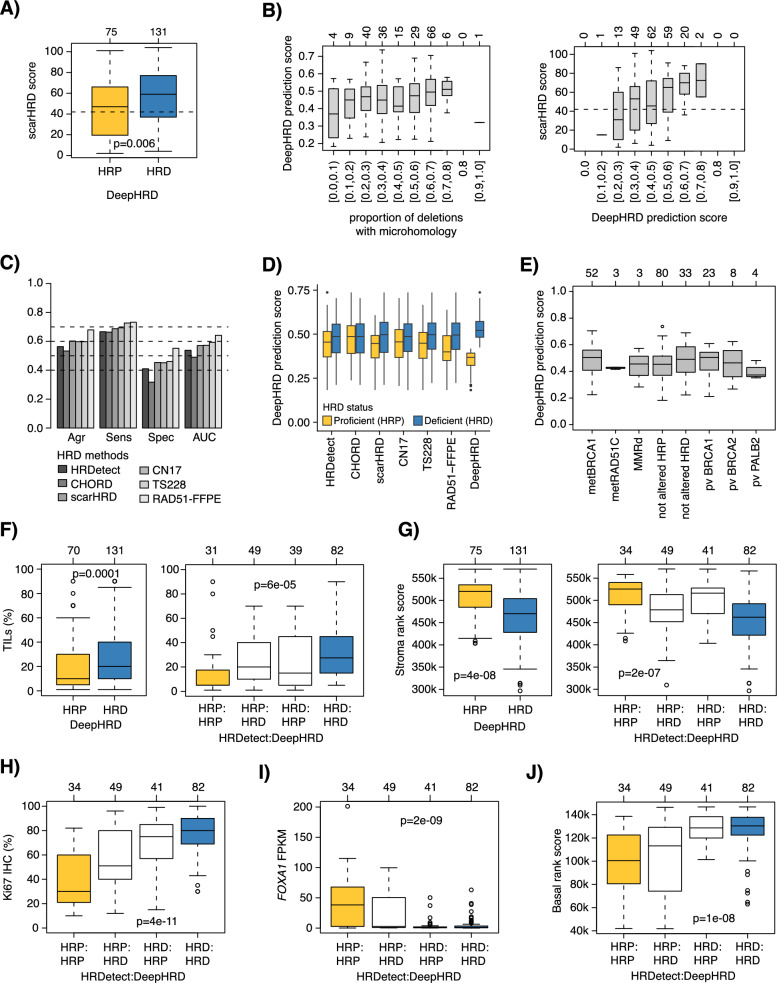
Concordance and discordance for DeepHRD classification. **A** scarHRD scores versus DeepHRD classes. Horizontal line corresponds to the scarHRD classification threshold of 42. Two-sided p-value computed using Wilcoxon’s test. **B** Left: binned proportion of deletions with microhomology versus DeepHRD prediction score. Right: binned DeepHRD prediction scores versus scarHRD scores. **C** Classification metrics for comparison between DeepHRD results and the other HRD methods as reference. Agr: agreement, sens: sensitivity, spec: specificity. **D** DeepHRD prediction scores versus HRD status for alternative HRD classification methods. **E** DeepHRD prediction scores versus HRD inactivation mechanisms and MMRd. Met: methylated promoter, pv: pathogenic variant (germline or somatic). HRD classification by HRDetect. **F** WSI TIL estimates versus DeepHRD classes (left) and combined HRDetect:DeepHRD classes (right). Two-sided p-values are calculated using Wilcoxon’s (left) or Kruskal–Wallis test (right). **G** Stroma metagene rank scores versus DeepHRD classes (left) and combined HRDetect:DeepHRD classes (right). Two-sided p-values computed using Wilcoxon’s (left) or Kruskal–Wallis test (right). **H** Ki67 IHC levels versus combined HRDetect:DeepHRD classes. **I** *FOXA1* FPKM levels versus combined HRDetect:DeepHRD classes. **J** Basal metagene rank scores versus combined HRDetect:DeepHRD classes. In H-I, two-sided p-values were calculated using Kruskal–Wallis test. Boxplot elements correspond to: i) center line = median, ii) box limits = upper and lower quartiles, iii) whiskers = 1.5 × interquartile range. Whenever present in boxplots, top axes indicate group sizes

DeepHRD was trained on The Cancer Genome Atlas (TCGA) cohort using scarHRD scores to distinguish between HRD and HRP tumors while balancing PAM50 subtype composition in the training and test cohorts [28]. In our cohort, DeepHRD showed generally the lowest agreements (kappa values) with other HRD methods (Fig. 2C), and prediction scores were significantly lower in nonBasal compared to Basal tumors (two-sided Wilcoxon’s test *p* = 0.002). DeepHRD classification was statistically associated with different scarHRD scores, although substantial overlap existed between HRP and HRD tumors (Fig. 6A). Moreover, we observed a Spearman correlation of 0.29 between scarHRD scores and DeepHRD prediction scores, illustrated by an increase in both scarHRD scores and proportion of deletions with microhomology with increasing DeepHRD prediction scores, although again with overlapping patterns (Fig. 6B). To analyze classification agreement more in depth we computed agreement, sensitivity, specificity, and area under the curve (AUC) scores for DeepHRD using the other methods as reference (Fig. 6C). This analysis demonstrated AUCs of 0.5–0.64 across methods, with the highest AUC observed between DeepHRD and RAD51-FFPE. For all six methods, DeepHRD prediction scores were generally higher for HRD cases, although substantial overlaps between HRP and HRD classes were observed (Fig. 6D). However, when tumors were stratified by proposed HRD inactivation mechanism, DeepHRD scores showed a less distinct pattern, even when compared to HRDetect HRP tumors (Fig. 6E).

To target specific concordance and discordance between DeepHRD and HRDetect classifications we investigated different molecular variables, as well as TME/TIME features. Bergstrom et al. [28] reported that spatial regions of breast cancer tissue predicted as HRD by DeepHRD were enriched for necrotic–neoplastic features and areas of high inflammatory cell density, including increased macrophage infiltration. To evaluate these TIME associations, we compared WSI-derived TIL estimates across both DeepHRD classifications and the combined HRDetect:DeepHRD categories. Tumors predicted as HRD by DeepHRD exhibited significantly higher TIL levels compared to HRP tumors (Fig. 6F). However, upon further stratification by HRDetect status, this difference appears primarily driven by the concordant HRP:HRP subgroup, which displayed notably lower TIL estimates (Fig. 6F). In opposite, a stroma gene expression metagene analysis showed highest rank scores in DeepHRD HRP tumors compared to HRD tumors, and more specifically in concordant HRP tumors and discordant HRD:HRP tumors (Fig. 6G). Next, DeepHRD HRD tumors showed higher Ki67 IHC levels than HRP tumors (two-sided Wilcoxon’s test p = 5e-05), and in agreement, further stratification by HRDetect showed the lowest proliferation in concordant HRP tumors and the highest in concordant HRD tumors (Fig. 6H). In TNBC, Ki67 levels differ significantly between Basal and nonBasal tumors (two-sided Wilcoxon’s test p = 6e-16). We therefore compared mRNA expression levels of *FOXA1* as a marker for nonBasal tumors, finding that concordant HRP tumors were defined by high *FOXA1* levels (i.e., a nonBasal feature), whereas concordant HRD tumors by low levels of this gene (Fig. 6I). Supporting this observation, a basal gene expression metagene showed an inverse rank score pattern, with high rank scores in concordant HRD tumors (Fig. 6J). Together, these results indicate that DeepHRD classification is influenced by both tumor intrinsic (e.g., proliferation) and extrinsic (TME/TIME) features, likely reflecting the composition of the original training cohort and setup.

### HRD methods and association with patient outcome after adjuvant chemotherapy

**Fig. 7:**
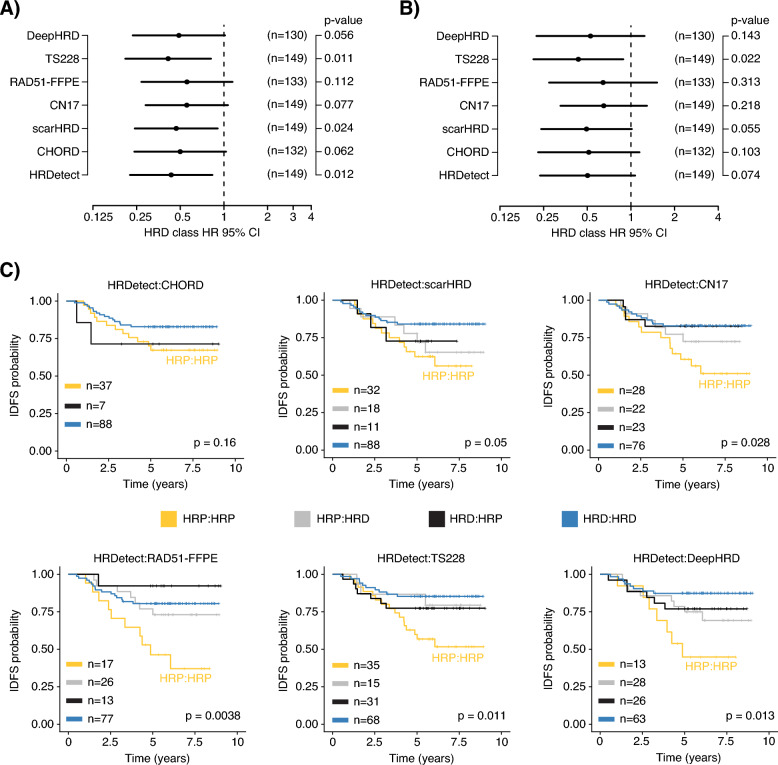
HRD methods and association with prognosis in chemotherapy-treated patients.** A** Forest plot of hazard ratios (HR) with 95% confidence intervals (CIs) obtained from univariate Cox regression per HRD method in chemotherapy-treated patients using invasive disease-free survival (IDFS) as clinical endpoint. For every method, HRP tumors were used as reference. Right axis shows corresponding p-values. Number of analyzed patients (n) indicated for each method.** B** Hazard ratios and 95% confidence intervals for the HRD class (HRP as reference) for each method based on multivariate Cox regression analysis using patient age, tumor size and grade, and lymph node status as covariates and IDFS as clinical endpoint. Right axis shows corresponding p-values. Number of analyzed patients (n) indicated for each method. **C** Kaplan–Meier plots of IDFS for combined HRD classes of HRDetect and the other six methods. P-values computed using the log-rank test. Group sizes indicated (n)

To investigate if the seven HRD classifiers differed in prognostic performance for 149 patients treated with standard-of-care adjuvant chemotherapy, we performed univariate Cox regression using invasive disease-free survival (IDFS) as clinical endpoint for each method (Fig. 7A). This analysis demonstrated that the overall prognostic performances were similar across methods on a group level (HRD vs. HRP) despite their variability in classification agreement, with hazard ratios around 0.5 for the HRD class. Next, we performed multivariate Cox regression analysis for each method using patient age at diagnosis (in years), tumor size (in mm), grade (Nottingham Histologic Grade, NHG), and lymph node involvement status (negative/positive) as covariates and IDFS as clinical endpoint (Fig. 7B). For all methods, hazard ratios were similar to those observed in the univariate analysis; the TS228 classification was statistically significant, while HRDetect and scarHRD were borderline non-significant (*p* = 0.074 and *p* = 0.055, respectively). Finally, we tested each HRD method with a multivariate Cox regression model using HRDetect as the only covariate and IDFS as clinical endpoint, finding that no HRD method provided significant independent prognostic information when adjusted for HRDetect status (all p > 0.05). Still, although limited by small sample numbers, an exploratory analysis showed that concordant HRP cases had the worst patient outcomes, while concordant HRD cases—except when classified with RAD51-FFPE—showed the best prognosis, indicating a potential prognostic enrichment of the combined classifications (Fig. 7C).

### Features of intermediate HRD tumors

For all investigated methods the binary HRD/HRP status reflects a classification threshold applied to, e.g., a GIS score, a probability, a correlation, or an estimated signature exposure. Consequently, there will invariably be tumors presenting borderline HRD/HRP profiles/scores that can be considered as an intermediate group. To explore the concept of an intermediate HRD phenotype we focused on HRDetect-classified tumors and defined three groups by subdividing HRP tumors into HRDetect-intermediate (HRD probability equal to or above 0.1 but lower than 0.7, n = 20) and HRDetect-low (HRD probability < 0.1, n = 76).

**Fig. 8:**
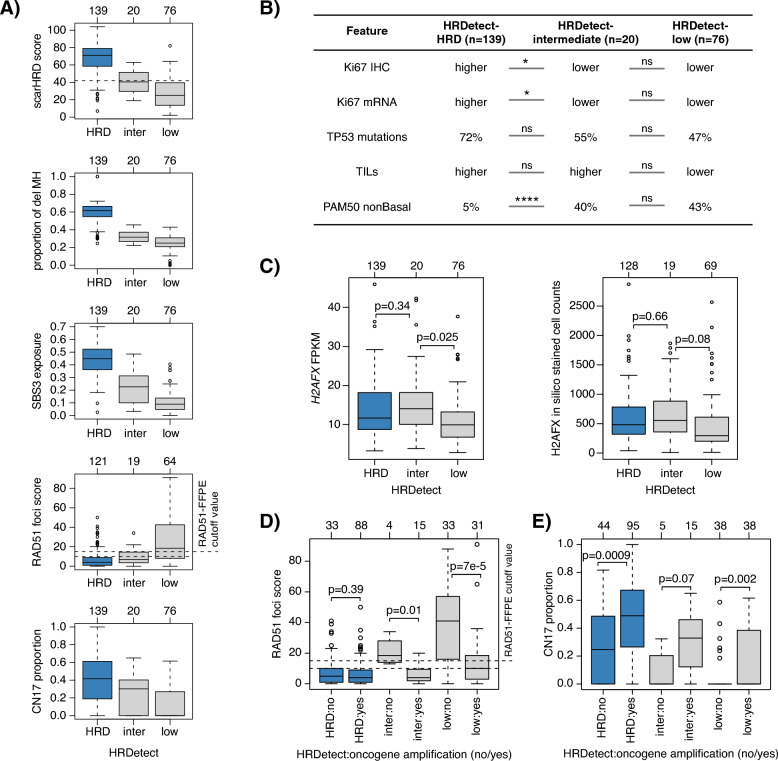
Features of intermediate HRD tumors. **A** From top to bottom: boxplots of scarHRD scores, proportion of deletions with flanking microhomology (del MH), exposure to SBS3, RAD51 foci scores, and CN17 proportions across the three HRDetect groups (HRD, inter: HRDetect-intermediate, low: HRDetect-low). **B** Summary of molecular features of interest for the three HRDetect groups. Two-sided tests performed using either Wilcoxon’s test or the Chi-square test (see Supplementary Figure S4 for details). ns: not significant (*p* > 0.05), *: *p* < 0.05, ****: *p* < 0.0001. **C** FPKM values (left) and stained cell counts estimated in silico (right) for the *H2AFX* gene across HRDetect groups. Two-sided p-values computed using Wilcoxon’s test. **D** RAD51 foci scores versus HRDetect classes combined with amplification status. Two-sided p-values computed using Wilcoxon’s test. **E** CN17 signature proportions versus the same combined classes as in D. Boxplot elements correspond to: i) center line = median, ii) box limits = upper and lower quartiles, iii) whiskers = 1.5 × interquartile range. In boxplots, top axes indicate group sizes

Considering the variables included in HRDetect (see [6]), the HRDetect-intermediate group showed, as expected, intermediate scarHRD scores, proportions of deletions with flanking microhomology, and exposure to the SBS3 mutational signature, but also intermediate RAD51 foci scores and intermediate exposure to the CN17 signature (Fig. 8A). Next, we analyzed IHC, RNA-sequencing, and WGS data to identify features differing between the three groups (summarized in Fig. 8B). Firstly, HRD tumors were more proliferative than both HRDetect-intermediate and HRDetect-low tumors, which showed equivalent Ki67 IHC and mRNA levels to each other (Supplementary Figure S4A). A similar pattern was observed for *TP53* mutation rates, with equal frequencies in HRDetect-intermediate and HRDetect-low tumors that were lower than those observed in HRD tumors, although not reaching statistical significance (Supplementary Figure S4B). In contrast, HRDetect-intermediate tumors were more similar to HRD tumors with respect to the TIME based on TIL levels, as infiltration was lower in HRDetect-low tumors (Supplementary Figure S4C). Notably, both the HRDetect-intermediate and the HRDetect-low groups contained ~ 40% of PAM50 nonBasal tumors, whereas only 5% of HRD tumors exhibited a nonBasal subtype (Supplementary Figure S4D). Consistently, this subtype proportion was accompanied by lower mRNA rank scores of a basal metagene and higher scores of a steroid response metagene in HRDetect-intermediate tumors compared to HRD tumors (Supplementary Figure S4E).

Next, we focused on aspects of DNA damage and on a proposed concept of oncogene-induced replication stress as a mechanism behind DNA damage [49, 50]. For the six genes (*NAT10*, *DDX27*, *ZNF48*, *C8ORF33*, *MOCS3*, and *MPP6*) reported by Lloblet et al. [51] as constituting an oncogene-induced replication stress mRNA signature, we observed no significant difference in FPKM levels between HRDetect-intermediate and HRDetect-low tumors (Wilcoxon test *p* > 0.05). In line with these findings, only 13 (6%) of the 219 DNA repair-associated genes showed differential expression between HRDetect-intermediate and HRDetect-low tumors, but not between HRD and HRDetect-intermediate tumors (unadjusted Wilcoxon’s test *p* < 0.05 and *p* > 0.05, respectively). The 13 genes were: *NEIL1*, *CCNH*, *SWI5*, *PDS5B*, *RAD54B*, *ABRAXAS1*, *RAD50*, *FAAP100*, *NUDT1*, *NUDT18*, *H2AFX*, *CHAF1A*, and *RPA4*. It should be noted that the absolute fold changes observed between test groups for these genes were lower, as no gene was significant after multiple testing correction (FDR-adjusted Wilcoxon’s test *p* > 0.05). Included in the 13 genes was *H2AFX* (H2A histone family member X, γH2AX), a proposed marker for double-strand DNA breaks [52], thus corroborating the in situ RAD51 foci score findings (Fig. 8C). To substantiate the *H2AFX* mRNA pattern we performed IHC staining for γH2AX and used a digital cell counting method developed by us [53] to estimate the number of positively stained cells, obtaining an overall Pearson correlation coefficient of 0.50 between estimates in a pattern consistent with the mRNA data (Fig. 8C). Lastly, we previously reported a higher prevalence of driver amplifications in HRDetect-intermediate tumors, with Cyclin E1 (*CCNE1*) located on chromosome 19q12 as the most amplified gene [5]. Based on screening 31 driver amplifications present in at least one tumor in the 235-sample cohort we found that more HRDetect-intermediate tumors (75%) were affected by amplifications than HRD (68%) or HRDetect-low (50%) tumors (Chi-square test *p* = 0.015). Moreover, HRDetect-intermediate tumors with amplifications showed lower RAD51 foci scores, indicating defective double-strand DNA repair, compared to non-amplified HRDetect-intermediate tumors (Fig. 8D). A similar significant RAD51 foci pattern was found for amplified HRDetect-low versus non-amplified HRDetect-low tumors, but not for HRD tumors. Differences in CN17 signature proportions were also observed between the same groups, with HRDetect-intermediate and HRDetect-low tumors with amplifications showing higher signature exposure compared to non-amplified cases (Fig. 8E).

## Discussion

In the current study we have performed a comprehensive comparison of HRD classifiers based on different data modalities in a large cohort of treatment-naïve primary TNBC tumors. Importantly, this comprehensive multi-omics cohort allows us to not only report concordance between methods, but also to analyze method discordance in detail regarding for instance HR inactivation mechanisms and TME features, in addition to testing the prognostic value of each method. Given the need for a consensus on how to define and measure HRD [16, 35, 36], this study provides valuable insights into several existing academic HRD classifiers.

Using the commercial FDA-approved Myriad myChoice CDx assay as reference in an exploratory analysis, we observed the highest classification agreement with the WGS-based CHORD and HRDetect methods, followed by scarHRD. Here it should be noted that while scarHRD is designed to approximate the Myriad GIS score, notable variability between the two computed scores was observed that may affect binary HRD classification agreement negatively in larger cohorts. Importantly, comparisons to the myChoice CDx assay performed in this study are exploratory in nature due to the small sample numbers, and further validation in larger cohorts is required to assess clinical equivalence. HRDetect, CHORD, and scarHRD also showed highest overall classification agreement to each other (with CHORD and HRDetect showing an almost perfect agreement) and they classified most *BRCA1*-deficient or *BRCA2/PALB2/RAD51C*-deficient tumors as HRD, with CHORD and HRDetect exceeding 99% accuracy. This high classifier overlap may be partly expected as the methods focus on similar genetic scar features (e.g., deletions with flanking microhomology). The major difference between CHORD and HRDetect in this study was that CHORD could not classify approximately 20% of tumors, presumably attributed to low tumor cell content coupled with limited WGS sequencing depth. Here, the more comprehensive HRDetect algorithm was able to classify all samples, including those samples that failed CHORD analysis. With respect to non-DNA-based methods, classification agreements were typically lower and more diverse.

Given the limited number of tumors here analyzed by the clinically-used Myriad myChoice CDx assay, we instead used HRDetect as a reference HRD classification in this research setting to further investigate concordance and discordance across methods. HRDetect was trained in breast cancer to specifically detect *BRCA1*- and *BRCA2*-deficient tumors using the somatic substitution, insertion/deletion and rearrangement patterns associated with *BRCA1*/*BRCA2* dysfunction that have been imprinted as genomic scars in the tumor genomes [6]. While the FDA-approved Myriad myChoice CDx assay would have been a preferred reference classification, the comprehensive WGS-based HRDetect predictor has been successfully applied to thousands of breast cancer samples from different academic institutions worldwide, demonstrating robustness versus different sampling and sequencing protocols and instruments [5, 6, 8–10, 44], despite requiring more preprocessing of WGS output (like signature fitting and copy number analysis). Still, discrepancies between different assays and HRDetect should be interpreted with caution, as using HRDetect as the comparator may oversimplify the biological complexity of HRD, favoring for instance a more *BRCA1*/*BRCA2*-like phenotype and not necessarily reflecting currently active HRD in a tumor. Importantly, different assays may capture distinct, and potentially complementary, aspects of the HRD phenotype. The analytical performance of DNA-based HRD assays is typically sensitive to sample tumor purity (the proportion of tumor cells relative to normal cells) and tumor heterogeneity (the range of tumor cells contributing to the HRD phenotype relative to other tumor cells) [17]. Consistently, the proposed limit of detection for the Myriad GIS score is ~30% tumor DNA content (www.myriad.com). In line with this, we observed lower tumor cellularity in “false negative” HRD tumors by scarHRD (classified as HRP by scarHRD) compared to HRDetect, an algorithm that includes multiple features besides SCNAs [6]. An association with lower tumor cell content was also observed for tumors that could not be classified by CHORD, likely indicating lower mutational burden given the sequencing depth of the analyzed samples. Interestingly, tumors classified discordantly by CHORD (HRP) and HRDetect (HRD) showed similar tumor cell content to concordant HRD tumors, but had substantially lower mutational burden. This may point to the impact of specific classification thresholds in CHORD or suggest that the two methods capture partially distinct biological features of HRD. Beyond tumor cell content, discordance between HRDetect and both scarHRD and CN17 also appeared influenced by segmentation performance, particularly the number and size of ASCAT-derived segments. Together, these findings highlight the importance of adequate sampling, such as macrodissection to ensure sufficient tumor cell content, as well as the impact of sequencing depth and key preprocessing steps, including segmentation, on the performance of DNA-based HRD classifiers.

The current study illustrates that HRD classification based on functional assessment of the number of RAD51 foci also faces challenges. Comparisons between RAD51 foci scores and prototypical DNA scars associated with HRD, as well as HRDetect results and explorative observations on distant metastatic relapse rates, suggest that adopting a lower foci cutoff could improve classification alignment with DNA-based methods. However, while lowering the foci cutoff improved method agreement with HRDetect, it did not increase the proportion of *BRCA1*- or *BRCA2*-deficient tumors classified as HRD by the RAD51-FFPE assay. It should be acknowledged that a limitation of our analyses is the use of FFPE TMAs for the RAD51-FFPE assay, as tissue cores may not fully capture the heterogeneity present in the entire tumor piece/section.

For the mRNA-based TS228 classifier, discrepant cases relative to the HRDetect classification were mainly observed in the PAM50 Basal subtype, without any consistent pattern of differences in broader transcriptional programs among the discrepant class combinations. In contrast, concordant HRP tumors showed typical traits of nonBasal TNBC like high expression of *FOXA1* and lower proliferation, whereas concordant HRD tumors were exclusively of the Basal phenotype. Given the reported association between HRD and a Basal phenotype in TNBC [5], these results illustrate the importance of cohort composition when training gene expression classifiers based on relative expression patterns. Here, it should be noted that the TS228 signature was trained in a breast cancer cohort comprising all clinical subgroups, without accounting for underlying molecular subtype [27]. In particular, gene expression-based centroid classifiers, like PAM50 and TS228, have repeatedly been shown to be sensitive to the composition of the training and test cohorts based on their need for gene centering [54, 55]. This well-recognized limitation of centroid-based classifiers, together with the marked biological differences between Basal and nonBasal TNBC, may explain the concordance and discordance observed between TS228 and HRDetect in our cohort. Specifically, HRD classifiers trained in a general breast cancer or even general TNBC context may capture mRNA expression patterns that are not specific to HRD status, which can limit their reproducibility in independent cohorts with different biological composition. An alternative approach would be to develop mRNA-based HRD predictors tailored specifically to Basal tumors, as this also represents the predominant patient subset with this genetic phenotype.

Overall, the image-based DeepHRD classifier showed the lowest agreement with other methods. DeepHRD was trained on H&E images from TCGA breast tumors of all clinical and molecular subtypes, using an HRD classification defined by scarHRD [28]. In our cohort, the classification agreement between scarHRD and DeepHRD was not notably different from that observed for other methods. Although DeepHRD was trained with balanced numbers of HRD and HRP cases across PAM50 subtypes, it combines tumors with potentially distinct tumor-intrinsic and -extrinsic characteristics (similar to the TS228 mRNA-based approach), which may affect both training performance and generalizability, particularly in specific molecular or clinical subgroups. For example, the morphological features of an HRD tumor, including tumor and TME/TIME characteristics, may differ between Basal TNBC and ER-positive/HER2-negative tumors. Our deeper analyses of concordance and discordance between DeepHRD and HRDetect in TNBC indicate that HRP concordance is typically observed for nonBasal tumors with lower proliferation levels, lower TIL levels, and higher stromal content, while concordant HRD tumors are typically of the Basal subtype with higher tumor proliferation. It remains unclear whether these observations reflect i) higher classification weight of classical tumor-intrinsic aspects of proliferative high-grade tumor cells like tubule formation, nuclear pleomorphism, and mitotic count visible in WSI data; ii) biases in training cohort composition or labeling; or iii) inherent differences in the TME between nonBasal and Basal tumors. Here, new image-based HRD classification methods should be explored, trained within more stringently defined molecular cohorts, like Basal TNBC, where HRD classification is most relevant. Finally, it should be acknowledged that differences such as in pre-analytical pathology parameters or image acquisition, as discussed by Marra et al. [56], could also confound our DeepHRD results compared to those of Bergstrom et al. [28], representing aspects that are difficult to address.

While a binary classification into HRP or HRD simplifies clinical decision making, tumors with an “intermediate” HRD phenotype are found in all classification methods, illustrating the need for a consensus definition of HRD [16]. In our cohort, the 8.5% of tumors with an HRDetect-intermediate phenotype showed both similarities and differences to HRDetect HRD and HRDetect-low tumors regarding specific gene mutations, proliferation scores, TIME status, and molecular subtypes. HRDetect-intermediate tumors showed intermediate RAD51 foci scores and *H2AFX* expression, both markers of DNA repair. However, RNA-sequencing data did not reveal clear expression differences in DNA repair–related genes across HRD, HRDetect-intermediate, and HRDetect-low groups. Notably, many HRDetect-intermediate tumors with oncogene amplification exhibited lower RAD51 foci scores, consistent with HRD, a pattern also observed in HRDetect-low tumors with similar amplifications. This was further reflected by the emergence of a CN17 signature in HRP (HRDetect-low) tumors with oncogene amplification. Together, these findings may help explain discrepancies observed with the SCNA-based CN17 method, suggesting that it may represent a less specific “genomic scar” of HRD compared with features such as deletions with flanking microhomology. Together, these observations provide some support to the hypothesis of oncogene-induced replication stress as a mechanism behind DNA damage [49, 50], although relevant only in a small number of TNBCs.

Limitations apply to the current study. Foremost, this study cannot address the key questions of how to definitively characterize or measure HRD, both important aspects that need to be addressed in order to enhance the clinical utility of HRD assessment in breast cancer [16]. The low number of cases analyzed with Myriad myChoice CDx (due to cost and tissue accessibility) hinders a more comprehensive comparison between the academic methods and an FDA-approved assay. Consequently, comparative results shown here should be considered exploratory, requiring validation in larger cohorts to prove clinical equivalence. Moreover, sample numbers limit the HRDetect-intermediate analyses, warranting further investigations based on the preliminary findings reported here. It should also be acknowledged that, while the non-FFPE DNA-based and RNA-based classifiers in this study all relied on nucleic acids extracted from the same tumor piece, this freshly preserved tissue specimen is different from the clinical FFPE tissue used for the TMA (RAD51-FFPE) and whole-slide (Myriad myChoice CDx and DeepHRD) methods. Finally, due to the retrospective nature of the cohort, no patient received adjuvant therapy with platinum-based agents or PARP inhibitors, in line with national treatment guidelines at the time. This limits the clinical interpretation of observed discordance between HRD classification methods in the context of more HRD-directed therapies. Interestingly, despite variability in classification across methods, survival analyses among patients receiving adjuvant chemotherapy (primarily FEC-based) showed similar overall prognostic performance for the different methods. This suggests that all methods capture a broader chemosensitive tumor phenotype, encompassing not only prototypical HRD tumors but also those with more general DNA repair deficiencies and higher proliferative activity, consistent with HRD arising through diverse alterations, several of which likely remain to be defined. Regarding classification concordance and discordance relative to the HRDetect comparator method and patient outcomes after adjuvant chemotherapy, patients with concordant HRP tumors consistently showed the poorest outcomes across all methods. In contrast, those with concordant HRD tumors had the most favorable outcomes, except when classified by the RAD51-FFPE method. Interpretation of discordant groups was less consistent, with outcomes varying between methods, possibly reflecting limited sample size and influence of individual cases. This variability highlights the need for analyses in larger clinical cohorts to enable more definitive conclusions.

In summary, to enhance the clinical utility of HRD assessment, a consensus is needed regarding both the definition of HRD and the methodologies used to evaluate it [16]. A critical component of this task is to elucidate the concordance and discordance among HRD classification methods derived from different data modalities, and to determine whether these classifications translate into meaningful differences in patient outcome. In this context, the present study provides new insights into the mechanisms underlying agreement and disagreement between HRD classifiers across data types, as well as their respective prognostic performances in patients receiving adjuvant chemotherapy. Moreover, our findings underscore the need for rigorous optimization of core data processing procedures and threshold parameters to ensure consistency, comparability, and reproducibility across analytical platforms.

## Methods

### Ethics approval and consent to participate

Patients were enrolled in the Sweden Cancerome Analysis Network – Breast (SCAN-B) study (ClinicalTrials.gov ID NCT02306096) [57, 58], approved by the Regional Ethical Review Board in Lund, Sweden, or the Swedish Ethical Review Authority (registration numbers 2009/658, 2010/383, 2012/58, 2013/459, 2014/521, 2015/277, 2016/541, 2016/742, 2016/9944, 2018/267, 2019–01252, and 2024–02040-02). All patients provided written informed consent prior to enrolment, including to publish information about sex and age. Inclusion and exclusion criteria for SCAN-B patients are outlined in NCT02306096. Patient gender was not considered as an inclusion or exclusion criteria for the study. All analyses were performed in accordance with patient consent and ethical regulations and decisions. This study conformed to the principles of the Helsinki Declaration.

### SCAN-B patient cohort

This study is based on a 235-sample SCAN-B TNBC patient cohort representative of the Swedish population reported by Staaf et al. [5], where participants were enrolled between 2010 and early 2015 in the Skåne healthcare region in South Sweden. Briefly, all tumor tissue used in this study was collected at time of surgery prior to any treatment start, i.e., representing treatment-naïve specimens. The main chemotherapy regimen for patients treated with adjuvant chemotherapy in this cohort was FEC-based (combination of 5 fluorouracil, epirubicin, and cyclophosphamide) ± a taxane in 96% of cases [5]. Due to the retrospective nature of the cohort with long-term follow-up, no patient received adjuvant therapy including platinum-based agents or PARP inhibitors in accordance with national treatment guidelines at the time. All included patients were analyzed by WGS (aligned to GRCh37) providing HRDetect calls, RNA-sequencing (available as fragments per kilobase million, FPKM, values [59]), and *BRCA1* and *RAD51C* promoter methylation profiling [5]. For this cohort, we assembled additional extensive clinicopathological data, patient follow-up data, H&E WSI TIL rates, molecular mRNA classifications (including PAM50 subtypes [39] and TNBCtype subtypes [40, 60]), and gene expression rank scores for eight biological metagenes (originally described by Fredlund et al. [47]) from either the study by Staaf et al. [5] or Aine et al. [41]. Exposure to SBS signatures were obtained from the study by Aine et al. [61], while exposure to structural rearrangement signatures were collected from Staaf et al. [5]. Classification of tumors as *BRCA1*-deficient or *BRCA2/PALB2/RAD51C*-deficient by germline or somatic pathogenic variants or promoter hypermethylation was obtained from [5]. No patients were tested by commercial, clinically approved, gene expression-based assays in a routine diagnostic setting as these were not included in national treatment guidelines during the inclusion period. Complete and extended tumor-level data can be found in Table S1 from Aine et al. [41]. A cohort summary regarding clinicopathological variables for the 235 cases is included in Supplementary Table S1.

### HRDetect classification

HRDetect classes were obtained from Staaf et al. [5]. HRD tumors were defined based on an HRDetect probability > 0.7. HRDetect classes are available in Supplementary Table S1.

### scarHRD HRD classification

scarHRD HRD scores were calculated based on reported segmented ASCAT data (available from [5]) using R scripts available from the scarHRD project’s GitHub [18]. Tumors with summarized HRD scores > 42 were called as HRD, otherwise HRP. scarHRD classes are available in Supplementary Table S1.

### Myriad Genetics HRD classification

Myriad myChoice CDx analysis was performed on 18 selected cases with available FFPE tissue blocks from the SCAN-B cohort. The analysis was performed through the Myriad partner laboratory in Denmark and HRD status and HRD scores (GIS scores) were obtained according to standardized protocols for the Myriad assay [62]. Myriad GIS scores are available in Supplementary Table S1.

### Copy number signature HRD classification

Copy number signature proportions per tumor for 25 signatures defined by Steele et al. [19] were calculated using segmented ASCAT data and code available from COSMIC v3.4 (https://cancer.sanger.ac.uk/signatures/cn/). Tumors were classified as HRD based on the CN17 signature exposure, as this signature has previously been associated with HRD [19]. Tumors with a CN17 exposure > 0 were called as HRD, otherwise HRP. As we observed considerable variability in CN17 HRD frequency depending on ASCAT segment sizes, we filtered original ASCAT SCNA segments from [5] to only retain segments > 500 Kbp prior to CN signature fitting for the main analysis. CN17 scores are available in Supplementary Table S1.

### CHORD HRD classification

CHORD [22] HRD classification was performed by first combining the CaVEMan (single nucleotide variants, SNVs) and Pindel (indels) VCF (Variant Call Format) data available from [5] into one common SNV VCF file per sample with Bcftools “concat" with option “-a" [63]. After the merge, only variants tagged PASS were retained. The structural variants (SVs) BRASS VCF files were used as is. HRD status was predicted with CHORD using the merged SNV and BRASS SV files as input separately for each sample. Reference genome was GRCh37 and option “-include_non_pass’’ was used to retain all variants in the BRASS VCF in CHORD prediction (BRASS does not tag variants PASS/noPASS and the PASS tag is required by CHORD). For a sample to be classified as HRD, a computed p-value equal to or higher than 0.5 and at least 50 indels were required. Of the 235 tumors, 188 were classified by CHORD as either HRD or HRP, while 47 tumors (20%) could not be determined. CHORD classes are available in Supplementary Table S1.

### RAD51-FFPE-based HRD classification

RAD51-FFPE-based HRD classifications were obtained for 204 tumors from Kramer et al. [46]. An additional 12 tumors were analyzed but classified as non-informative due to different quality issues. Briefly, the RAD51-FFPE test (co-immunofluorescence staining of RAD51 and geminin) was performed on tissue microarray (TMA) cores from matched FFPE blocks for SCAN-B patients as described [32, 46]. TMA construction for the SCAN-B cohort has been previously described [61]. Geminin-positive cells were identified on each core, and 30 or more geminin-positive cells were selected at random for the calculation of a RAD51 score (percentage of geminin-positive cells with two or more RAD51 foci). A RAD51-FFPE score lower than or equal to 15% was used to classify TNBC samples as HRD [32, 64]. RAD51-FFPE HRD classes are available in Supplementary Table S1.

### Gene expression-based HRD classification

Gene expression-based HRD classification (TS228) was performed using the 228-gene HRD nearest centroid classifier (comprising one HRD centroid and one HRP centroid) reported by Jacobson et al. [27] (obtained from Table S4 in [27]). The TS228 HRD signature was developed using 857 TCGA breast tumors representing diverse clinical and molecular subgroups. The model was trained using multinomial elastic net regression on expression profiles from 572 tumors to distinguish between different forms of HRD and HR proficiency, while the remaining TCGA samples were used for testing. Training labels (HRD/HRP) were inferred through clustering of mutational signature profiles derived from matched whole-exome sequencing data, although the original study did not explicitly provide the training and test labels [27]. Prior to classification using SCAN-B FPKM data, an offset of 0.1 was added, the data were log2-transformed and mean-centered across the 235 tumors. For each sample, Pearson correlation to each centroid was computed in addition to an HRD score as outlined by Jacobson et al. Classification was performed using a specific correlation cutoff (Pearson correlation > 0.05) applied to the HRD centroid values. This specific cutoff was selected as it optimized sensitivity, specificity and agreement with HRDetect classification (Supplementary Figure S3). HRD classifications are available in Supplementary Table S1.

### H&E image-based HRD classification using DeepHRD

Digital H&E WSI of FFPE sections from matched FFPE tumor blocks for 206 of the 235 tumors were used as input to the DeepHRD AI algorithm [28]. H&E-stained slides were digitized as previously described [65]. DeepHRD was executed within a singularity/apptainer container using the pre-trained breast cancer FFPE model from the original publication, with GPU acceleration enabled. Predictions were carried out using the following parameters indicated by the authors for the pre-trained model: –BN_reps 10 –preprocess –stainNorm –generateDataSets –predict5x –pullROIs –predict20x. HRD classifications are available in Supplementary Table S1.

### Gene expression analyses

In differential gene expression analysis, FPKM data were offset by + 0.1 followed by log2 centering. Two-group comparisons were performed using Wilcoxon’s test, while multi-group comparisons were performed using the Kruskal–Wallis test. FDR adjustment for multiple testing was performed using the R *p.adjust()* function. Clustering was performed using Pearson correlation and Ward.D linkage on median-centered log2-transformed FPKM data using the R pheatmap package (v1.0.12). Upset plots were created using the UpSetR R package (v1.4.0).

### H2A histone family member X immunohistochemistry analysis

H2A histone family member X, γH2AX, immunohistochemistry was performed on matching TMAs (five blocks) comprising two 1 mm cores per tumor. Staining was performed using an antibody from CellSignaling (cat. #9718) with a 1:500 dilution, Dako PT-Link Buffer pH9 pretreatment, Dako EnVision Flex K8010 visualization and 30 min incubation time. In total, cores from 216 patients could be evaluated using the automated TMArQ (Tissue microarray MArker Quantification) software [53] with default settings. For each tumor, the mean positive stained cell count from available cores was computed.

### Statistics and survival analyses

Cohen’s kappa was calculated using the *cohen.kappa()* function in the R package psych (v2.5.3). Survival analyses were performed in R version 4.4.2 using the survival package (v3.7.0) with invasive disease-free survival (IDFS) as clinical endpoint. Survival curves were compared using Kaplan–Meier estimates and the log-rank test. Univariate and multivariate Cox regression were performed to estimate hazard ratios using the *coxph()* R function. In multivariate analysis, tumor size (in mm), patient age at diagnosis (in years), Nottingham Histological Grade, and lymph node status (negative/positive) were included as covariates.

## Supplementary Information

Below is the link to the electronic supplementary material.

Supplementary Material

Supplementary Material 1

Supplementary Material 2

## Data Availability

The SCAN-B primary tumor genomic dataset supporting the conclusions of this article is available in open repositories as described in the original studies. Briefly, the SCAN-B WGS data used in this study, originally reported by Staaf et al. [5], are available from [https://data.mendeley.com/datasets/2mn4ctdpxp/3]. All SCAN-B RNA-sequencing data used in this study, originally reported by Staaf et al. [59], are available from [https://data.mendeley.com/datasets/yzxtxn4nmd/3].
