# Supplementary material for: Comprehensive comparison of homologous recombination deficiency predictors in early-stage triple-negative breast cancer
Source: Breast Cancer Res. 2026 Jun 25;28:115. doi: 10.1186/s13058-026-02325-5 (PMC13295736; doi:10.1186/s13058-026-02325-5)
Supplement: Supplementary file 1 — Supplementary Material [file 13058_2026_2325_MOESM1_ESM.pdf]

A)

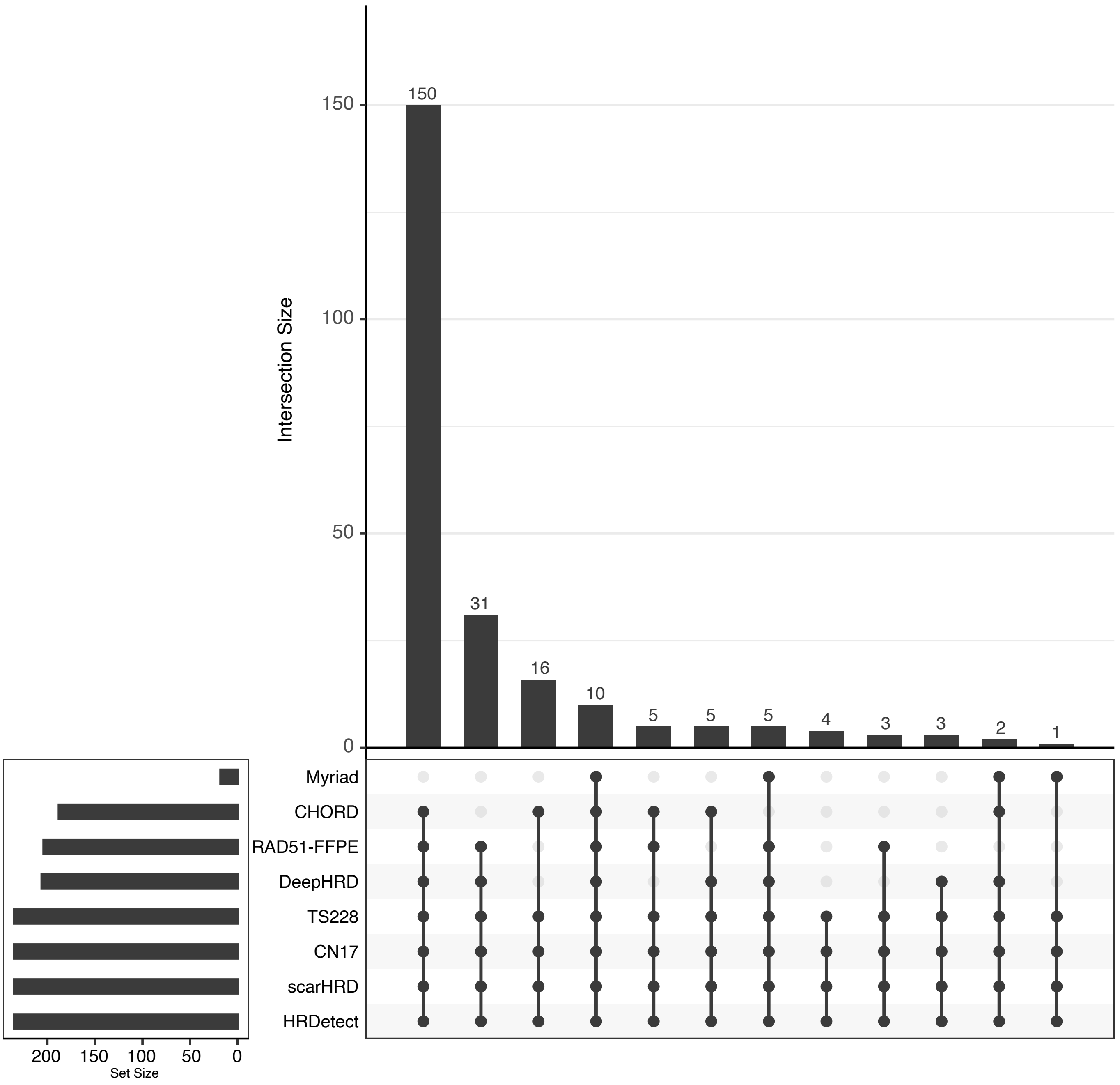

Supplementary Figure S1. HRD method sample overlap and characteristics of the HRDetect HRP tumor PD31144a. (**A**) Sample overlap illustrated by an upset plot for the different HRD methods. (**B**) Raw ASCAT segmentation plot of logR data (SCNA) and BAF for sample PD31144a. Data for the figure were obtained from Staaf et al. Nature Medicine 2019. The SCNA and BAF plots of PD31144a show a pattern of alterations prototypical for *BRCA1*-deficient tumors. (**C**) Circos plot of WGS alterations detected in PD31144a. Data for the figure were obtained from Staaf et al. Nature Medicine 2019. The tumor shows distinct features of *BRCA1*-deficiency in addition to MMRd and was accordingly found to carry *BRCA1* promoter hypermethylation with corresponding low level of expressed *BRCA1* mRNA. Circos plot depicting from outermost rings heading inwards: Karyotypic ideogram outermost. Base substitutions next, plotted as rainfall plots (log10 intermutation distance on radial axis, dot colours: blue, C>A; black, C>G; red, C>T; grey, T>A; green, T>C; pink, T>G). Ring with short green lines, insertions; ring with short red lines, deletions. Major copy number allele ring (green, gain), minor copy number allele ring (red, loss), Central lines represent rearrangements (green, tandem duplications; red, deletions; blue, inversions; grey, interchromosomal events). The circos plot for PD31144a shows the prototypical pattern of a *BRCA1*-deficient tumor if the SBS signature proportion is disregarded, including a higher number of deletions with flanking microhomology and a higher proportion of structural rearrangement signature 3 (RS3). The SBS signature proportion is heavily skewed towards SBS6 (s6) due to the MMRd also present in the tumor.

B)

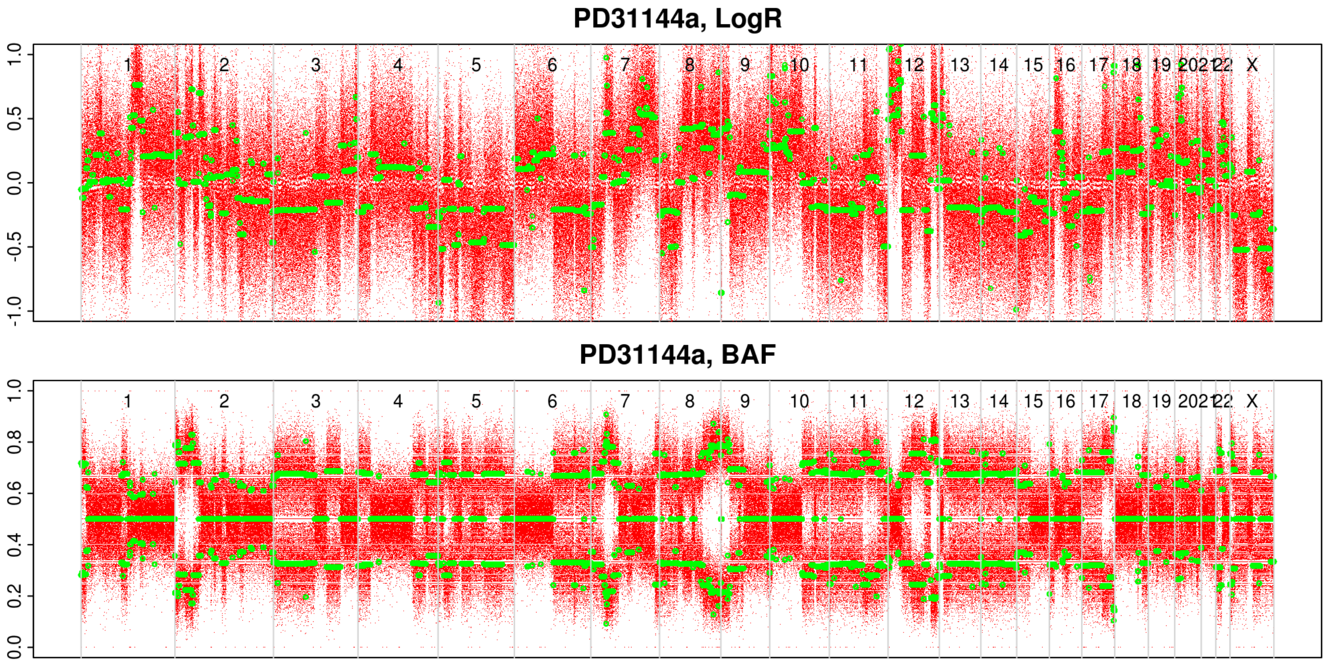
PD31144a, LogR
PD31144a, BAF
1 2 3 4 5 6 7 8 9 10 11 12 13 14 15 16 17 18 19 20 21 22 X

C)

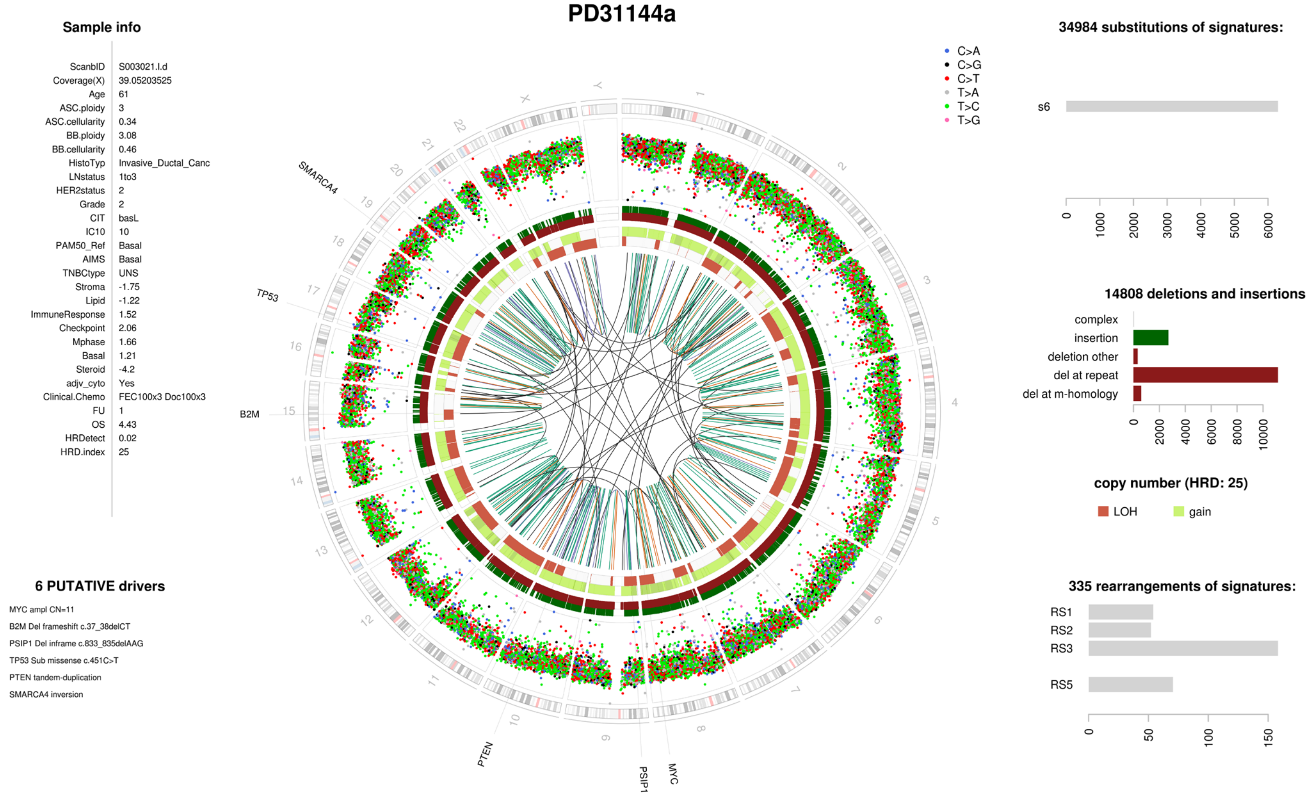
PD31144a
Sample info
ScanbID S003021.l.d
Coverage(X) 39.05203525
Age 61
ASC.ploidy 3
ASC.cellularity 0.34
BB.ploidy 3.08
BB.cellularity 0.46
HistoTyp Invasive_Ductal_Canc
LNstatus 1to3
HER2status 2
Grade 2
CIT basL
IC10 10
PAM50_Ref Basal
AIMS Basal
TNBCtype UNS
Stroma -1.75
Lipid -1.22
ImmuneResponse 1.52
Checkpoint 2.06
Mphase 1.66
Basal 1.21
Steroid -4.2
adjv_cyto Yes
Clinical.Chemo FEC100x3 Doc100x3
FU 1
OS 4.43
HRDetect 0.02
HRD.index 25
6 PUTATIVE drivers
MYC ampl CN=11
B2M Del frameshift c.37_38delCT
PSIP1 Del inframe c.833_835delAAG
TP53 Sub missense c.451C>T
PTEN tandem-duplication
SMARCA4 inversion
C>A
C>G
C>T
T>A
T>C
T>G
SMARCA4
TP53
B2M
PTEN
PSIP1
MYC
34984 substitutions of signatures:
s6
14808 deletions and insertions
complex
insertion
deletion other
del at repeat
del at m-homology
copy number (HRD: 25)
LOH
gain
335 rearrangements of signatures:
RS1
RS2
RS3
RS5

A)

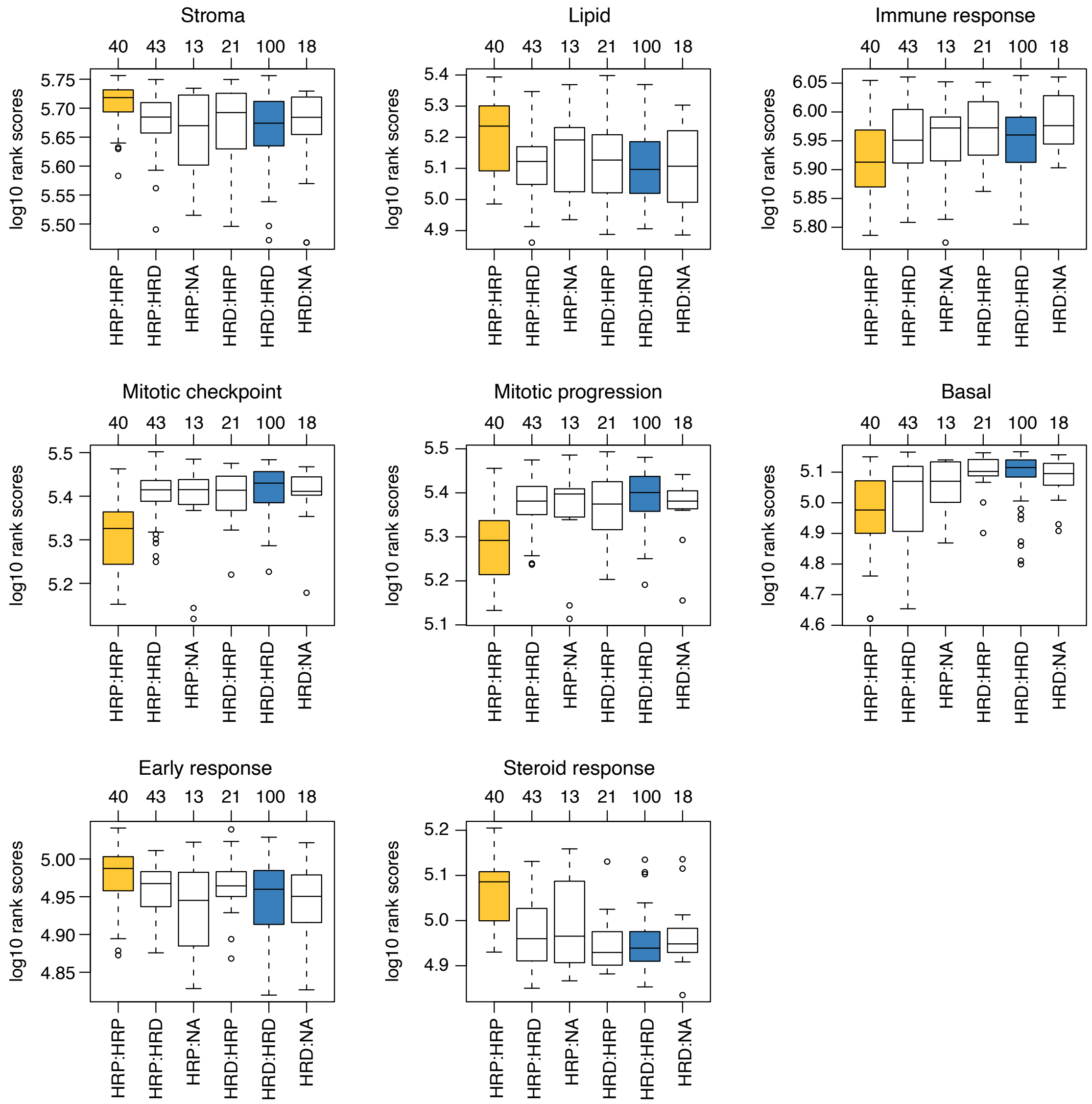

B)

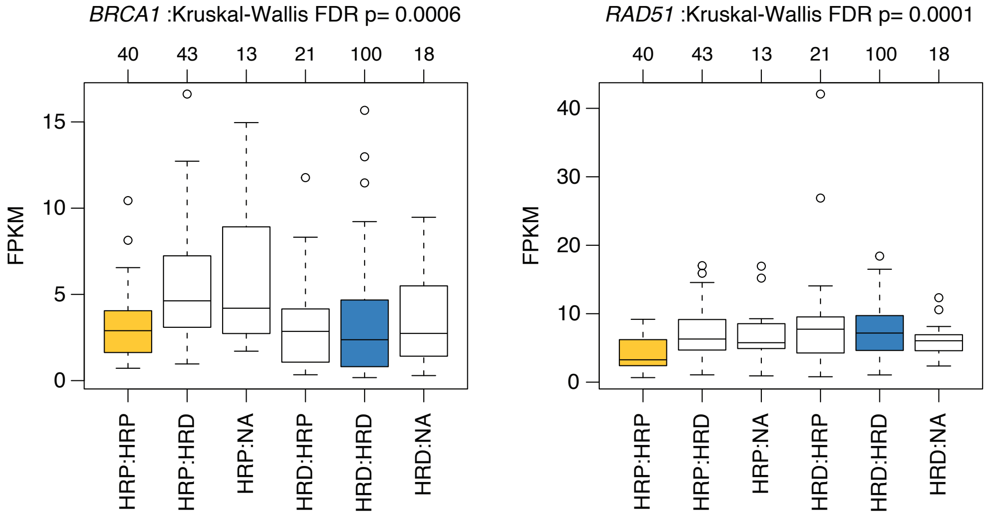

Supplementary Figure S2. Gene expression differences for combined HRDetect and RAD51-FFPE classifications. (**A**) Log10 transformed rank scores for eight biological metagenes versus combined HRDetect and RAD51-FFPE (HRDetect:RAD51-FFPE) classifications. NA: RAD51 classification not available. (**B**) Gene expression (FPKM) levels of *BRCA1* and *RAD51* versus the same combined classification groups.

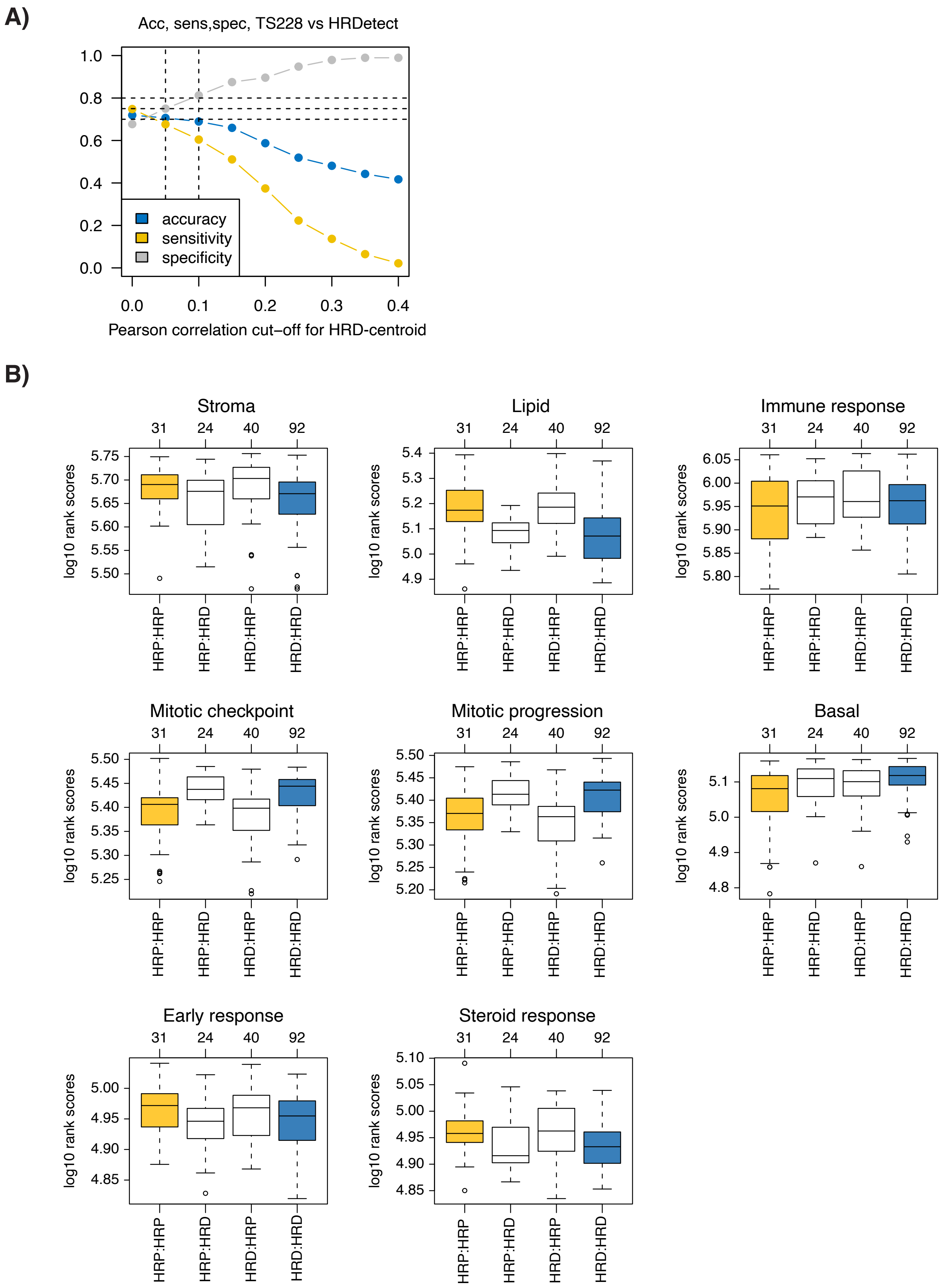

Supplementary Figure S3. Gene expression differences for combined HRDetect and gene expression based HRD classification (TS228). (A) Selection of correlation cut-off for TS228, using the HRD-centroid, based on comparison to HRDetect. A cut-off of 0.05 was selected as it optimized sensitivity, specificity, and accuracy. **(B)** Log10 transformed rank scores for eight biological metagenes versus combined HRDetect and gene expression classification by TS228 (HRDetect:TS228) classifications.

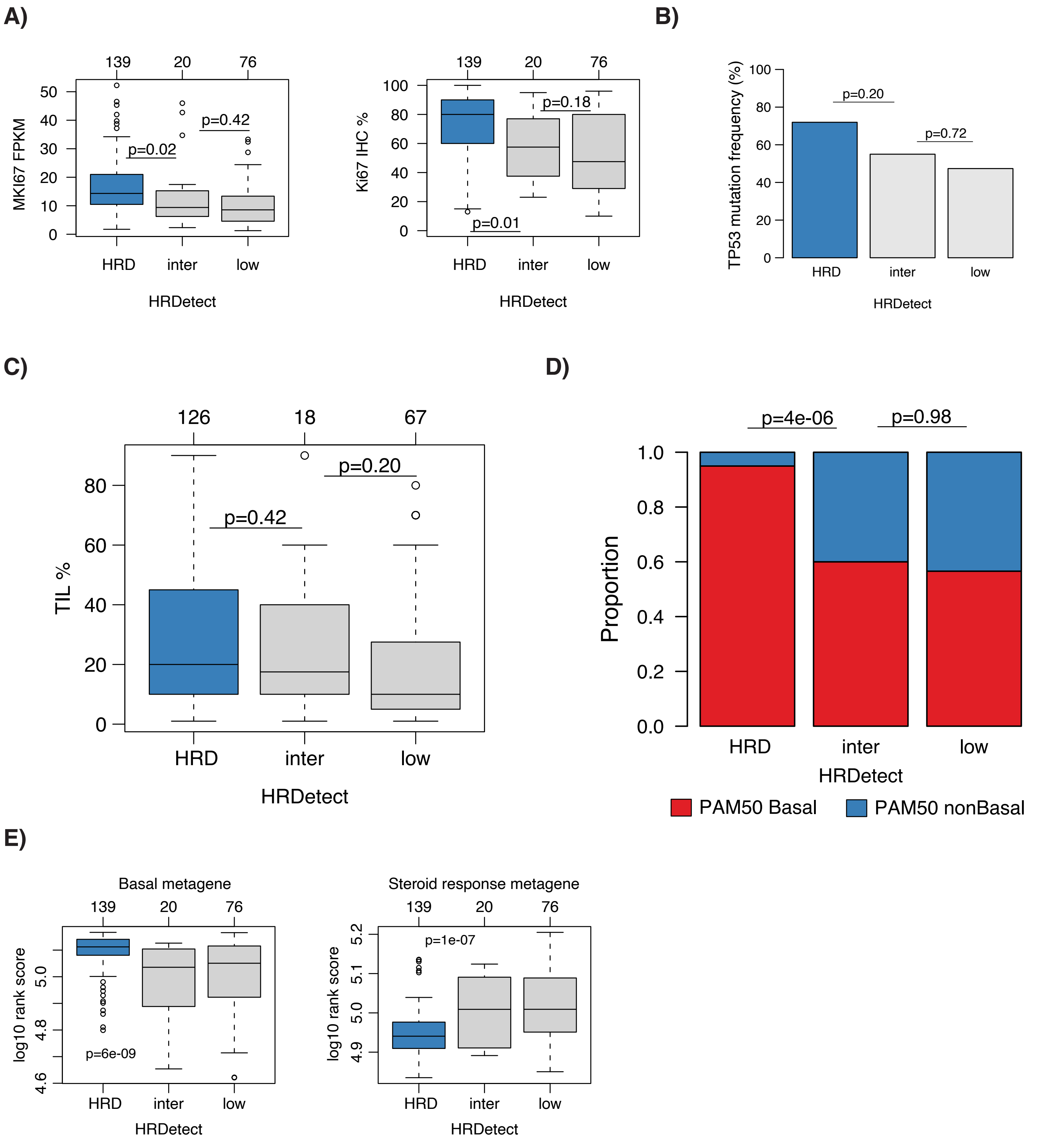

Supplementary Figure S4. Analyses of HRDetect-intermediate tumors. (A) Left: Ki67 mRNA levels (*MKI67*, FPKM) and Ki67 IHC levels (right) versus HRDetect groups (inter: HRDetect-intermediate, low: HRDetect-low). Two-sided p-values calculated using Wilcoxon's test. **(B)** *TP53* mutation frequency in HRDetect groups. Two-sided p-values calculated using the Chi-square test. **(C)** Whole slide TIL percentages versus HRDetect groups. Two-sided p-values calculated using Wilcoxon's test. **(D)** PAM50 proportions in HRDetect groups. Two-sided p-values calculated using the Chi-square test. **(E)** Gene expression rank scores for a Basal metagene (left) and a steroid response metagene versus HRDetect groups. Two-sided p-values calculated using Kruskal-Wallis test.
